# Stimuli-Responsive Nanomaterial-Based Biosensor Structures for Wound Care: pH, ROS, and Temperature Sensing Strategies

**DOI:** 10.3390/mi17030306

**Published:** 2026-02-28

**Authors:** Anita Ioana Visan, Adrian Birnaz, Irina Negut

**Affiliations:** 1 National Institute for Laser, Plasma and Radiation Physics (INFLPR), Atomiştilor 409, 077125 Magurele, Romania; anita.visan@inflpr.ro; 2 Center for Nanotechnology and Nanosensors, Department of Microelectronics and Biomedical Engineering, Technical University of Moldova, 168 Ștefan cel Mare Avenue, MD-2004 Chisinau, Moldova; adrian.birnaz@mib.utm.md

**Keywords:** smart wound biosensors, stimuli-responsive nanomaterials, theranostic wound care

## Abstract

Chronic and infected wounds remain a major clinical challenge due to their dynamic microenvironments and the lack of real-time diagnostic feedback in conventional dressings. Recent advances in stimuli-responsive nanomaterial-based biosensors have enabled the development of smart wound-care systems capable of continuous monitoring and on-demand therapeutic intervention. This review systematically summarizes progress in nanomaterial-enabled wound biosensing strategies, with a focus on pH, reactive oxygen species, and temperature nanosensors, which serve as key indicators of infection, inflammation, and healing status. We discuss the sensing mechanisms and functional roles of diverse nanomaterials. A particular focus is placed on emerging multimodal and theranostic platforms which integrate biochemical and physical sensing with controlled drug release, photothermal or photodynamic therapy, and redox regulation. These systems represent a shift from passive wound monitoring toward closed-loop, adaptive wound management. Also, future perspectives are outlined, highlighting the convergence of nanomaterials, self-powered electronics, and intelligent data processing as a pathway toward personalized and precision wound care.

## 1. Introduction

Effective wound healing depends on regulated biochemical and physiological conditions at the wound site. Disruption of this balance, as commonly observed in chronic wounds such as diabetic ulcers and infected traumatic injuries, leads to delayed healing, persistent inflammation, and increased risk of severe complications [1]. Traditional wound assessment relies largely on visual inspection and intermittent clinical evaluation, which often fail to detect early pathological changes occurring at the molecular and physicochemical level. As a result, there is a growing need for technologies capable of monitoring wound microenvironment parameters in situ and in real time [2]. Experimental evidence shows that key alterations in wound pH, oxidative stress, and metabolic activity precede visible clinical symptoms, limiting the effectiveness of conventional assessment approaches [3]. As a result, there is a growing need for technologies capable of monitoring wound microenvironment parameters in situ and in real time to enable earlier diagnosis and more informed therapeutic intervention [4].

Among the most informative indicators of wound status, pH, reactive oxygen species (ROS), and local temperature have emerged as key biomarkers reflecting infection, inflammation, and tissue perfusion [1,5,6]. Experimental studies have shown that deviations from the mildly acidic pH characteristic of healing wounds toward alkaline values are strongly associated with bacterial colonization and chronicity [2].

ROS represents another critical determinant of wound healing outcomes. While moderate ROS levels support antimicrobial defense and angiogenesis, excessive ROS accumulation causes oxidative stress, prolonged inflammation, and tissue damage [4]. Recent experimental nanoplatforms have leveraged ROS-responsive materials to both sense and regulate oxidative stress in infected wounds [5].

Local temperature variation represents a clinically relevant parameter, as elevated wound temperature often precedes visible signs of infection or inflammation, whereas abnormally low temperatures may indicate ischemia or impaired perfusion. Experimental studies using skin-mounted and soft-surface temperature sensors have demonstrated that localized temperature changes can be detected earlier than macroscopic symptoms and correlate with inflammatory responses and altered blood flow [7]. Advances in nanomaterial-based thermoresistive bionanosensors have enabled accurate, flexible, and continuous temperature monitoring on soft biological surfaces [8].

Recent progress in nanomaterials has been central to these advances. Metallic nanoparticles, carbon-based nanostructures, polymeric nanocomposites, quantum dots (QDs), and two-dimensional materials provide high surface area, tunable surface chemistry, and unique electrical or optical responses to environmental stimuli. When embedded in hydrogels, electrospun fibers, or thin films, these materials enable stimuli-responsive nanosensors that convert pH, ROS, or temperature changes into visible, fluorescent, or electrical signals without the need for complex instrumentation [3,4]. Importantly, many of these systems are designed for direct wound contact, offering biocompatibility, conformability, and real-time responsiveness under physiologically relevant conditions [9,10,11].

Recent reviews have addressed smart wound dressings, wearable biosensors, and nanomaterial-enabled therapeutic systems. For example, several reviews [12,13] discussed intelligent nanomaterial dressings with antibacterial and ROS-modulating functions, while others reviewed flexible and wearable biosensing platforms for health monitoring [14,15]. However, these works either emphasize general smart-dressing strategies, wearable electronics, or therapeutic nanomaterials independently. In contrast, the present review provides a focused and mechanistically structured analysis centered specifically on pH, ROS, and temperature as interrelated wound biomarkers, integrating nano–bio interfacial sensing mechanisms with transduction principles and theranostic platform design. By concentrating on these three parameters, which are strongly linked to infection, inflammation, and healing progression, this review aims to provide a focused assessment of sensing mechanisms, material design strategies, and current progress, while identifying opportunities for future development of clinically translatable wound-monitoring technologies. In addition to diagnostic sensing, this review also highlights emerging nanomaterial-based theranostic platforms that integrate real-time biomarker monitoring with controlled drug release, photothermal or photodynamic therapy, redox modulation, and closed-loop wound management strategies.

## 2. Fundamentals of Wound Healing and the Need for Real-Time Monitoring

### 2.1. Biological Stages of Wound Healing

**Figure 1:**
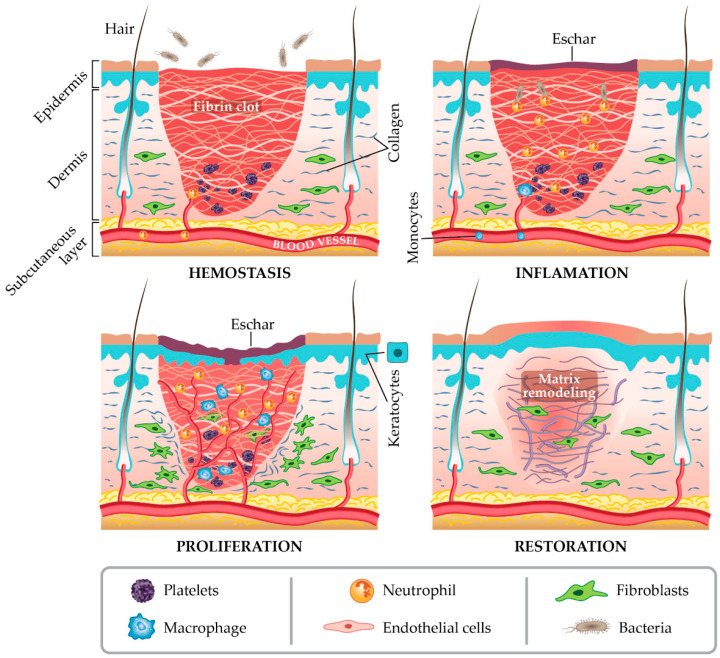
The four stages of wound healing [18].

Wound healing is a complex and highly coordinated biological process that restores the structural and functional integrity of injured tissue. It occurs through four interdependent and overlapping phases: hemostasis, inflammation, proliferation, and remodeling (Figure 1). During hemostasis, blood clotting rapidly seals the wound and forms a temporary extracellular matrix that serves as a scaffold for cellular migration. The inflammatory phase follows, characterized by the recruitment of immune cells such as neutrophils and macrophages, which eliminate debris and pathogens while releasing cytokines that regulate tissue repair. The proliferative phase involves fibroblast activation, collagen deposition, angiogenesis, and granulation tissue formation, whereas the remodeling phase strengthens the new tissue through collagen reorganization and maturation over time [16,17].

**Figure 2:**
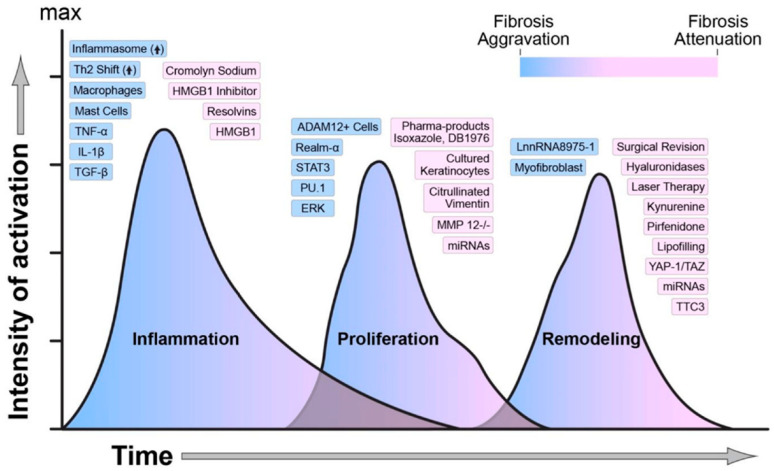
Regulators of wound healing and fibrosis. The progression, overlap, and intensity of activities within each phase of wound healing are tightly controlled by a complex network of molecular, cellular, and mechanical factors. These elements collectively determine the rate and quality of tissue repair as well as the extent of fibrotic response. The accompanying illustration depicts the interactions among these regulatory components, where blue shading represents activation, and pink indicates the suppression or reduction of fibrotic processes [19].

Wound healing phases are important to be understood due to the fact that they provide knowledge on how disturbances in infections, comorbidities, or a lack of correct care can postpone the process of wound healing. These also help the progress of therapeutic tactics, applied at each step in the process of wound healing. Figure 2 provides an overview of the molecular mediators and key biomarkers which regulate each phase, offering an understanding of their roles.

### 2.2. Biochemical and Physiological Markers in Wound Healing

The biochemical and physiological microenvironment of a wound provides critical insights into the underlying biological processes driving tissue repair. During the wound-healing cascade, a complex interplay of biomolecular signals, physicochemical gradients, and cellular activities governs the transition across hemostasis, inflammation, proliferation, and remodeling phases. The dynamic behavior of these parameters, such as pH, ROS, cytokine expression, temperature, and oxygen levels, reflects the wound’s current state and healing trajectory. Monitoring these markers in real time provides a quantitative understanding of tissue status, offering early warning of infection, ischemia, or chronicity that may not be visible through clinical examination alone [20].

The table below (Table 1) summarizes the key biochemical and physiological markers associated with each stage of wound healing, their biological functions, highlighting their clinical and experimental relevance.

Among these indicators, pH plays a pivotal role in regulating enzymatic activity, microbial colonization, and oxygen diffusion. The pH of a healing wound typically ranges between 5.5 and 6.5, an acidic environment that supports fibroblast activity, collagen cross-linking, and angiogenesis. Acidic pH values also inhibit bacterial proliferation and reduce the activity of proteolytic enzymes that degrade extracellular matrix components. By contrast, chronic wounds frequently exhibit alkaline pH values (≥7.3), which disrupt cell migration and increase susceptibility to infection. This shift often correlates with biofilm formation and excessive exudate, leading to a self-perpetuating cycle of delayed repair [20].

ROS, including superoxide anions, hydrogen peroxide, and hydroxyl radicals, serve as double-edged regulators of healing [24]. At moderate concentrations, ROS act as crucial signaling molecules that stimulate angiogenesis, cell proliferation, and defense mechanisms [25]. However, in chronic wounds or under conditions of poor vascularization, ROS production exceeds antioxidant capacity, resulting in oxidative stress and extensive cellular damage [25]. This imbalance leads to lipid peroxidation, protein oxidation, and DNA fragmentation, impairing keratinocyte migration and tissue regeneration [24]. Recent biosensing approaches incorporate ROS-responsive nanomaterials and electrochemical transducers that enable continuous redox state assessment at the wound site, providing a valuable tool for tracking inflammation levels [24].

Temperature is another vital physiological parameter reflecting perfusion, inflammation, and infection. Normal wound sites exhibit slight hyperthermia due to increased metabolic activity and vascularization during healing. A localized rise in temperature (≥2 °C above adjacent healthy tissue) often indicates bacterial infection or inflammation, while hypothermic zones can suggest ischemia, necrosis, or impaired circulation [11]. Smart wound dressings embedded with thermal sensors now allow continuous, non-invasive temperature mapping, enabling clinicians to detect infection before visible symptoms develop [11].

Cytokines and growth factors are fundamental biochemical markers coordinating cellular signaling throughout healing. Early in the inflammatory phase, cytokines such as IL-1β, IL-6, and TNF-α activate immune cell recruitment and matrix remodeling [26]. During proliferation, growth factors including VEGF, TGF-β, and platelet-derived growth factor (PDGF) promote fibroblast proliferation, collagen synthesis, and angiogenesis [27,28]. Dysregulated or prolonged cytokine expression is a hallmark of chronic wounds, contributing to persistent inflammation and tissue degradation [29]. Advances in microfluidic and immunosensor technologies have enabled multiplexed detection of cytokines and growth factors, facilitating a more holistic assessment of wound status [29].

Oxygenation is another indispensable physiological factor in the wound-healing process. Adequate oxygen supply supports collagen cross-linking, fibroblast proliferation, and bacterial killing via oxidative pathways. Hypoxia, often seen in diabetic or pressure ulcers, leads to reduced cellular energy production and impaired angiogenesis. The ability to continuously monitor tissue oxygen tension (pO_2_) and oxygen saturation (SpO_2_) at the wound interface is thus critical. Modern biosensors utilizing optical fluorescence quenching and electrochemical Clark-type mechanisms can measure oxygen concentration in situ, enabling targeted oxygen therapy and improving healing outcomes [22].

In recent research on wound healing and infection monitoring, infection-associated metabolic byproducts such as trimethylamine (TMA) and UA have gained attention as informative biomarkers for early infection detection. TMA is a volatile organic compound produced predominantly by bacterial metabolism, especially under anaerobic conditions, and its presence may indicate bacterial colonization or the formation of biofilms in wounds, features often seen in chronic infected wounds. Quantifying TMA with wearable sensors offers the potential for rapid, non-invasive infection detection, enabling clinicians to intervene promptly and prevent further progression of infection [23,30]. UA, a purine metabolism end product, also serves as a meaningful wound biomarker. Elevated UA levels in wound exudate are frequently associated with oxidative stress and inflammation, both of which are hallmarks of infected or poorly healing wounds. Higher UA concentrations can further exacerbate inflammation by promoting ROS generation and activating immune responses. Monitoring UA in wound fluids through biosensing platforms can therefore provide valuable insight into the inflammatory and metabolic state of the wound environment, offering clinicians a more nuanced understanding of healing dynamics [23].

Other biochemical markers, including glucose, lactate, and bacterial metabolites, also play key roles in characterizing wound health and healing dynamics. Elevated glucose concentrations within wound tissue or exudate are frequently observed in individuals with diabetes, where impaired glucose regulation leads to delayed healing, reduced angiogenesis, and increased infection risk [21]. In contrast, lactate, a product of anaerobic metabolism, serves as both an energy substrate for regenerating tissues and a potential indicator of tissue hypoxia; its accumulation may signify inadequate oxygen supply within the wound microenvironment [31,32].

Furthermore, bacterial metabolites such as ammonia and volatile organic compounds (VOCs) are typically elevated during infection, reflecting increased microbial activity and metabolic imbalance [33].

Collectively, these biochemical and physiological markers provide a comprehensive portrait of the wound microenvironment. Their real-time monitoring through integrated biosensing systems enhances diagnostic accuracy but also enables adaptive wound management, where treatment strategies dynamically adjust based on sensor feedback. Such intelligent, data-driven approaches represent a significant step forward in personalized wound care, offering the potential to reduce chronic wound prevalence, hospital stays, and healthcare costs worldwide.

### 2.3. Why Traditional Wound Assessment Methods Are Insufficient?

Traditional wound assessment methods, including visual inspection, manual measurement, and photographic documentation, still remain the most common clinical practices for evaluating wound healing [34]. While these approaches are simple, inexpensive, and widely accessible, they possess inherent limitations that make them subjective, inconsistent, and often clinically inadequate for accurate, real-time wound evaluation.

Clinicians have relied on visual grading systems to estimate wound size, color, exudate levels, tissue type [34]. Yet, such assessments are qualitative and heavily dependent on clinical experience and also leading to significant inter- and intra-observer variability [35]. Different clinicians may record markedly different interpretations of the same wound, particularly regarding granulation tissue percentage, necrosis extent, or infection severity. These inconsistencies compromise reproducibility and hinder longitudinal comparisons of wound progression [34].

Manual measurements using rulers, acetate tracings, or wound planimetry have also been standard practice. Yet, these techniques often assume simple geometric shapes and cannot accurately capture the irregular, 3D morphology of most chronic or traumatic wounds. Studies have demonstrated that two-dimensional measurements tend to overestimate wound area by up to 44%, introducing systematic errors in healing rate estimations [36]. Moreover, manual measurement requires direct contact with the wound surface, increasing the risk of contamination, patient discomfort, and potential disruption of the fragile healing tissue [37].

In addition to geometric limitations, manual wound depth assessment is notoriously unreliable. Depth is often estimated using cotton-tipped applicators or probes, which provide only rough approximations and cannot capture microstructural changes within the wound bed. This approach also fails to differentiate between viable and necrotic tissue at different depths. Consequently, clinicians lack objective data to evaluate wound volume reduction, one of the most accurate indicators of healing dynamics [37].

Another major drawback of traditional assessment methods is their irregular and static nature. Wound evaluation typically occurs only during scheduled clinical visits, providing a limited number of discrete observations over the healing process. However, wound physiology is dynamic, with continuous fluctuations in temperature, oxygenation, pH, and biochemical markers. These temporal variations often precede visible clinical signs of infection or necrosis, meaning that traditional assessments can miss early pathological changes, delaying intervention and worsening patient outcomes [38].

The lack of quantitative biomarkers in conventional methods further limits their diagnostic value. Current wound scoring scales such as the PUSH Tool, Bates-Jensen Wound Assessment Tool (BWAT), and DESIGN-R, primarily rely on visual cues and subjective descriptors. A recent review found that none of these scales demonstrated strong content validity or inter-rater reliability, highlighting the urgent need for standardized, objective wound measurement frameworks [38].

Additionally, photographic documentation, while helpful for tracking wound evolution, often suffers from lighting inconsistencies, color distortion, and scaling errors, limiting its clinical accuracy. Without advanced imaging calibration or 3D reconstruction, such photographs fail to convey essential depth and texture information. Even when digital photography is standardized, it still lacks real-time functional data. Temperature, moisture, or biochemical gradients are crucial for understanding the wound microenvironment [38].

## 3. Biosensors—Overview

A biosensor is a device which combines a biological element, such as an enzyme, antibody, or microorganism, with an electronic system to produce a measurable signal [39]. The electronic component detects, processes, and transmits information about physiological changes or the presence of chemical or biological substances in the environment [40].

Biosensors can vary in shape and size but are designed to detect even very small amounts of specific pathogens, toxic compounds, or pH changes [41].

A typical biosensor consists of the following components:

(a) Analyte—the substance to be measured,
(b) Bioreceptor—the biological element that recognizes the analyte,
(c) Transducer—converts the biological response into an electrical signal,
(d) Electronics—amplifies and processes the signal, and
(e) Display—shows the final, readable output.

**Figure 3:**
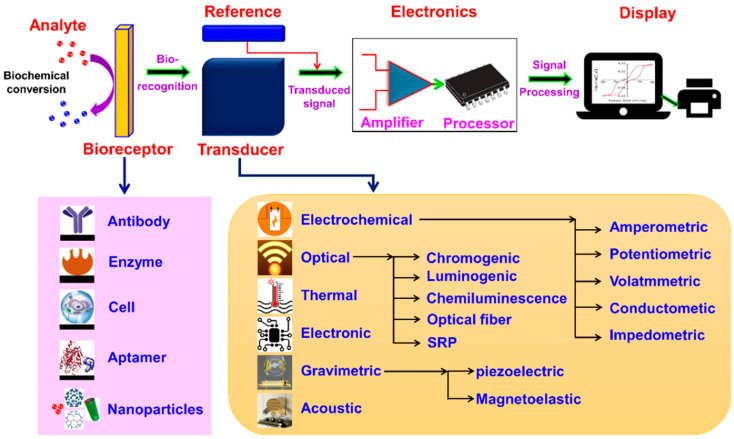
A typical biosensor consists of four main components: a bioreceptor, transducer, electronic system, and display unit. The analyte is the target substance (e.g., glucose, ammonia, alcohol, lactose). The bioreceptor, such as enzymes, cells, aptamers, DNA/RNA, or antibodies, specifically recognizes the analyte, producing a detectable change (e.g., in light, heat, pH, charge, or mass) known as biorecognition. The transducer converts this biological interaction into a measurable signal (electrical or optical), a process called signalization. Transducers are commonly classified as electrochemical, optical, thermal, electronic, or gravimetric. The electronic unit amplifies and processes the signal, converting it into digital form, while the display presents the results in numerical, graphical, or visual formats for user interpretation [42].

In Figure 3 is presented a schematic diagram of a typical biosensor.

### 3.1. Classification of Biosensors

To design a highly efficient and reliable biosensor system, both static and dynamic parameters must be considered [43]. These performance criteria help optimize biosensor functionality for commercial and practical applications:

(a) Selectivity is a key parameter that determines a biosensor’s ability to specifically recognize and detect the target analyte in the presence of other substances or contaminants. Choosing a bioreceptor with high specificity ensures accurate detection in complex samples [44].
(b) Sensitivity refers to the minimum detectable concentration of an analyte, often in the range of nanograms to femtograms per milliliter (ng/mL or fg/mL). A highly sensitive biosensor can accurately identify trace levels of analytes with minimal sample preparation [43].
(c) Linearity reflects the degree to which the biosensor’s output signal corresponds proportionally to the analyte concentration. Greater linearity enhances the accuracy and reliability of quantitative measurements [44].
(d) Response time is the duration required for the biosensor to achieve 95% of its final signal after exposure to the analyte. Faster response times improve real-time monitoring capabilities [45,46].
(e) Reproducibility indicates the biosensor’s ability to produce consistent results when analyzing the same sample multiple times. It encompasses precision (consistent readings) and accuracy (closeness to the true value) [42].
(f) Stability defines how well a biosensor maintains its performance over time under varying environmental conditions. It depends on factors such as the bioreceptor’s binding affinity, degradation rate, and resistance to external disturbances, particularly in applications requiring continuous monitoring [47].

**Figure 4:**
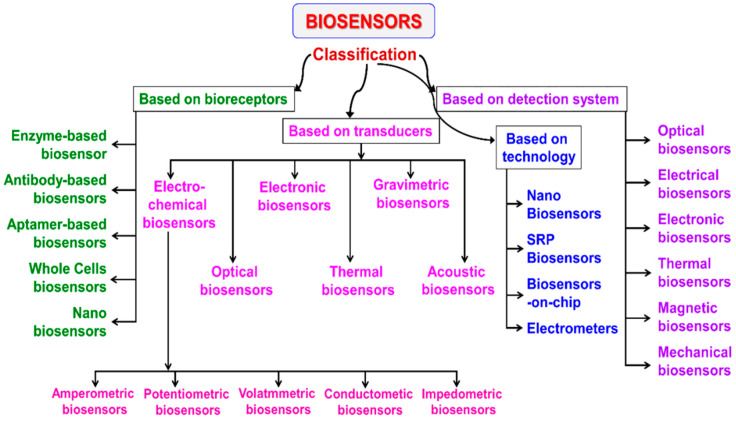
Classification of biosensors based on various bioreceptors and transducers used [42].

The classification of biosensors is derived from multiple scientific disciplines and relies on several distinct criteria. An overview of this classification framework is illustrated in Figure 4.

As previously noted, the bioreceptor serves as the central element in any biosensor design. Based on the type of bioreceptor employed, biosensors can be categorized into several main groups: (i) enzymatic biosensors, the most widely used type, which utilize enzymes as recognition elements [48]; (ii) immunosensors, known for their exceptional specificity and sensitivity, especially in medical diagnostics [49]; (iii) aptamer or nucleic acid-based biosensors, which exhibit strong selectivity toward microbial strains and nucleic acid-containing analyte [50]; and (iv) microbial or whole-cell biosensors, which use living cells to detect target substances [51].

A second major classification is based on the transduction principle. According to the transducer type, biosensors are divided into electrochemical (including potentiometric, amperometric, impedance, and conductometric), electronic, thermal, optical, and mass-sensitive (gravimetric) biosensors [52].

Another classification approach focuses on bioreceptor–analyte interactions, although this category is less extensive. Additionally, biosensors can be differentiated by the detection system employed, such as optical, electrical, thermal, mechanical, or magnetic detection, and by the technology platform used, including nanotechnology-based systems, surface plasmon resonance (SPR) sensors, lab-on-chip (biosensors-on-chip) devices, electrometer-based biosensors, and portable or deployable systems.

Biosensors can be classified in several ways based on what they measure or how they operate. The main categories include:

(a) Energy source—active and passive sensors
(b) Physical contact—contact and non-contact sensors
(c) Comparability—absolute and relative sensors
(d) Signal type—analog and digital sensors
(e) Detection method—physical, chemical, thermal, or biological sensors

(a) Active and passive biosensors

Active sensors require an external energy source to function (for example, microphones, thermistors, strain gauges, and capacitive or inductive sensors). These are also called parametric sensors, as their output depends on a measured parameter. Passive sensors, on the other hand, generate their own signal without the need for external power. Examples include thermocouples, piezoelectric sensors, and photodiodes, also known as self-generating sensors [53].

(b) Contact and non-contact biosensors

Contact sensors must physically touch the object or environment being measured, such as temperature probes. Non-contact sensors do not require direct contact and are typically based on optical, magnetic, or infrared principles, for example, IR thermometers and optical sensors [53].

(c) Absolute and relative biosensors

Absolute sensors, such as thermistors and strain gauges, measure a quantity on an absolute scale [54].

Analog sensors produce a continuous signal corresponding to the measured physical quantity. Common examples include thermocouples, resistance temperature detectors, and strain gauges. Digital sensors, by contrast, output discrete signals or pulses, encoders are a typical example [55].

(d) Based on signal detection

Sensors can also be categorized by the type of signal they detect: physical, chemical, thermal, or biological.

- Physical sensors: Measure physical quantities like force, acceleration, flow rate, mass, density, or pressure. Widely used in biomedical applications, they benefit from microelectromechanical systems (MEMS) technology for high precision and compact design [56].
- Chemical sensors: As defined by IUPAC, these devices convert chemical information (e.g., concentration or composition) into measurable signals. They are used for environmental monitoring, food and drug testing, pollution control, and clinical diagnostics [57].
- Thermal sensors: Measure temperature and convert it into an electronic signal. Common examples include thermocouples, thermistors, and RTDs [58].
- Biological sensors (Biosensors): Detect biological interactions such as antigen–antibody binding, DNA hybridization, enzyme reactions, or cell communication. These are widely used in medical diagnostics and biotechnology [59].

Recent reviews have addressed the classification of biosensors by both their biorecognition elements and transducer mechanisms to organize the rapidly expanding field. Gao et al. systematically outline biosensor structure and classification methods, emphasizing categories such as enzymatic, immunological, optical, thermal, and electrochemical sensors in the context of bio-manufacturing applications [22]. Chen et al. provide a broad overview of biosensor types, highlighting how different architectures and sensing principles enable diverse analytical applications [60]. Javaid et al. focus on the classification of nanophotonic biosensors, categorizing them into specific optical sensing modalities based on resonant structures and light-matter interactions [61].

### 3.2. Structural Design and Detection Mechanisms

Wearable wound-monitoring nanosensors are typically built upon multilayer flexible architectures, consisting of (1) a substrate, which provides mechanical support and flexibility; (2) a nanomaterial-based sensing layer, responsible for signal transduction; and (3) a protective encapsulation layer, which maintains biocompatibility and environmental stability [62].

Flexible substrates such as polydimethylsiloxane (PDMS), polyimide (PI), hydrogels, and textile fibers are favored for their elasticity and conformability to biological tissues. The sensing layers are enhanced using nanostructured materials like graphene, carbon nanotubes (CNTs), MXenes, and metallic nanoparticles, which offer high surface-to-volume ratios, excellent electrical conductivity, and tunable optical and chemical properties [63].

Hybrid nanocomposites, such as graphene–AuNP, MXene–polymer, and CNT–hydrogel systemsare increasingly being used to improve mechanical robustness, signal stability, and bio-interface compatibility [64].

These combinations allow the sensors to maintain accuracy under repetitive mechanical strain and moisture exposure typical of the wound microenvironment.

Wearable nanosensors are typically organized into three core layers:

1. Flexible substrate: Provides the mechanical backbone of the sensor, supporting structural flexibility and stretchability while ensuring intimate contact with irregular skin or wound surfaces [65].
2. Sensing layer: A nanomaterial-based active region responsible for converting biological or physicochemical stimuli (e.g., pH, temperature, cytokine concentration, or oxygen level) into measurable electrical or optical signals [66].
3. Encapsulation layer: Protects both the sensing interface and the wound bed from external contaminants, fluid interference, and mechanical abrasion, while maintaining biocompatibility and gas permeability to avoid skin irritation or wound maceration [66].

This multilayer structure ensures the device can withstand continuous deformation (bending, stretching, or twisting) during patient movement, while maintaining consistent signal output.

The substrate material plays a pivotal role in dictating the sensor’s conformability, breathability, and comfort. Commonly used flexible substrates include:

- PDMS: Exhibits excellent elasticity, transparency, and chemical inertness. Its tunable mechanical modulus allows PDMS-based sensors to conform to soft tissues, minimizing motion artifacts during wound monitoring [67].
- Polyimide (PI): Offers high thermal and mechanical stability, enabling high-performance nanoelectronic integration [68,69]. However, its relatively low moisture permeability may necessitate surface modifications for skin compatibility [70].
- Hydrogels: Mimic the softness and water content of biological tissue, providing superior comfort and biocompatibility [71]. Conductive hydrogels incorporating ionic nanofillers or metallic nanoparticles enable simultaneous sensing and healing promotion due to their bioactive properties [72].
- Textile fibers and nanofiber membranes: Allow for breathable, lightweight, and washable wearable designs. Electrospun nanofibers can be functionalized with graphene oxide or MXene nanoflakes to produce conductive, mechanically robust wound dressings with sensing capability [73].

At the “heart” of the nanosensor lies the active sensing layer, where nanomaterials play a decisive role in enhancing signal sensitivity, response speed, and selectivity.

Wearable nanobiosensors for wound monitoring rely on diverse signal transduction mechanisms that convert biochemical, biophysical, or environmental stimuli into electrical, optical, or thermal outputs. Each mechanism offers distinct advantages in terms of sensitivity, response time, and integration potential with flexible electronics (Table 2). The combination of these principles enables comprehensive, continuous, and non-invasive wound monitoring.

Next-generation wearable wound sensors are trending toward multimodal detection platforms, integrating two or more transduction mechanisms to achieve cross-validation of data and improved reliability.

Examples include:

- Electrochemical–optical hybrids, where colorimetric pH sensing is combined with amperometric glucose detection [89,90].
- Thermal–electrical composites, which correlate local temperature rise with impedance changes for infection identification [86,91].
- Triboelectric–electrochemical systems, which self-power biosensing circuits from patient motion [73].

AI-enhanced data fusion from multimodal sensors allows real-time pattern recognition, enabling predictive diagnostics in chronic wound care [92].

## 4. Nanomaterials in Wound Sensing and Healing

Wound care is rapidly evolving from passive coverage to interactive, sensing and therapeutic systems. Several core properties determine performance in wound sensing and healing:

(i) Biocompatibility and biodegradability: These are essential in order to avoid chronic inflammation, cytotoxicity and systemic accumulation; typically enhanced by using natural polymers (chitosan, alginate, cellulose) or green-synthesized metallic nanoparticles [74,93,94,95,96].
(ii) Electrical and ionic conductivity: Critical for electrochemical detection of pH, glucose, lactate, and for recording bioelectrical signals or delivering therapeutic electrical stimulation; provided primarily by carbon nanomaterials, metallic nanoparticles, MXenes, and conductive polymer networks [75,76,77,97].
(iii) Surface functionalization: Chemical modification with enzymes, antibodies, peptides or nucleic acids enables selective recognition of wound biomarkers (e.g., cytokines, matrix metalloproteinases, bacterial products), enhances dispersion, and tunes charge and hydrophilicity [76,78,79,98].

These properties reinforce the integration of nanomaterials into flexible, conformal dressings and wearable platforms capable of continuous monitoring and feedback-controlled therapy [75,77,78,82].

The convergence of these mechanisms with therapeutic functionalities (e.g., antibacterial action, ROS modulation, growth-factor delivery, analgesia and pro-angiogenic effects) positions nanomaterial-based nanosensors as central components of next-generation smart dressings and wearable wound-monitoring systems [2,6,9,13,14].

Nanomaterials, by the virtue of their size, high surface area, tunable chemistry, and unique electrical/optical properties, are central to this shift, enabling dressings that both monitor the wound microenvironment and actively promote repair [74,77,97]. Key classes include metallic, carbon-based, and polymeric nanomaterials, as well as QDs and MXenes, each contributing distinct sensing and healing functions.

### 4.1. Metallic Nanoparticles

Metallic NPs are widely used as dual-function antibacterial agents and sensing elements.

Silver, gold, zinc oxide and titanium dioxide exhibit strong antimicrobial activity and can be incorporated into membranes, hydrogels, or nanocomposite scaffolds to prevent infection and accelerate closure [93]. In addition to direct bactericidal effects, ZnO and other metal oxides can generate or modulate ROS, which is valuable for both disinfection and redox-responsive therapies when carefully controlled [9]. Their excellent electrical and optical properties allow integration into electrochemical and optical biosensors for analytes such as glucose, H_2_O_2_ and other metabolic markers [78].

In the study by Deng et al., a pain-relieving drug–loaded hydrogel dressing was developed for the treatment of infected burn wounds. The system was formulated by combining polyvinyl alcohol grafted with gallic acid (PVA-GA), sodium alginate grafted with 3-aminobenzeneboronic acid (SA-PBA), Zn^2+^ ions, and chitosan-coated borneol nanoparticles possessing anti-inflammatory and analgesic properties. These components formed a nanoparticle-loaded hydrogel network (PVA-GA/Zn^2+^/SA-PBA) through multiple physicochemical crosslinking interactions. The resulting hydrogel exhibited excellent properties, including strong adhesiveness, self-healing ability, shape adaptability, injectability, biodegradability, and conformity to irregular wound surfaces. It also demonstrated desirable biological functions such as pH-responsive drug release, antibacterial, antioxidant, anti-inflammatory, and pro-angiogenic activities. In a murine scald wound model, the hydrogel effectively inhibited Staphylococcus aureus infection and significantly reduced pain perception, as assessed by mouse grimace scale scores and hind paw lifting and licking behavior. These combined effects contributed to accelerated wound healing [99].

In the study by Lee et al., a chitosan-based composite hydrogel (SNPCHG) co-loaded with silver ions and nanoparticle-encapsulated epidermal growth factor provided sustained release of both agents and markedly improved closure of diabetic wounds in rats, achieving ~97% closure at day 14 with enhanced re-epithelialization and collagen deposition compared with commercial dressings [100].

In the work of Wei et al., a self-assembled mangiferin hydrogel encapsulating rhein-functionalized silver nanoparticles (Rh@Ag) showed strong bactericidal activity, mitigated inflammation, and significantly promoted re-epithelialization, collagen deposition, and angiogenesis in infected diabetic wounds, largely via antioxidant and pro-regenerative signaling (e.g., PI3K–Akt, ECM–receptor interaction) [101].

In the study by Liu et al., a versatile ZnAu nanodot–loaded hydrogel (ZAN@HG) released zinc ions and exhibited enhanced photothermal and fluorescent properties, enabling both rapid healing of MRSA-infected diabetic wounds and real-time visual indication of when dressing replacement was needed through fluorescence changes. The versatile nanocomposite wound dressing with zinc replenishment, and enhanced photothermal and fluorescent performance for accelerating methicillin-resistant Staphylococcus aureus-infected diabetic wound repair and indicating dressing replacement [102].

Green-synthesized metallic nanoparticles and metal–polymer composites further improve biocompatibility and safety, while enabling stimuli-responsive “intelligent” dressings that can monitor pH or infection markers and trigger drug release on demand [74,78,97].

### 4.2. Carbon-Based Nanomaterials

Carbon nanomaterials (graphene, graphene oxide, CNTs, carbon dots, graphene QDs, fullerenes, nanodiamonds) provide the electronic backbone of many smart dressings due to high conductivity, rich surface chemistry and tunable photoluminescence [75,76,79,103]. When embedded in hydrogels or polymer matrices, they confer:

- High electrical conductivity for electrochemical and piezoresistive sensing of pH, metabolites and mechanical deformation [75,76].
- Strong antibacterial and anti-oxidant activity, reducing biofilm formation and excessive inflammation [75,76,79].
- Intrinsic fluorescence (CDs, CQDs, GQDs) for optical and colorimetric detection of wound biomarkers (e.g., pH, glucose, urea, proteins), often readable by simple imaging or smartphone analysis [75].

In the work of Zaffar et al., an alginate–polyethylenimine hydrogel infused with fluorescent carbon dots and rose-petal extracellular vesicles formed a syringe-injectable dressing that selectively targeted Gram-negative bacteria, maintained low inflammation in diabetic rat wounds, and leveraged the intrinsic antimicrobial activity of carbon dots [104].

In the study by He et al., N-carboxyethyl chitosan/Pluronic F127 hydrogels incorporating carbon nanotubes produced a conductive, self-healing, adhesive dressing with strong in vitro and in vivo photothermal antibacterial activity and improved closure, collagen deposition, and angiogenesis in infected full-thickness skin wounds [105].

In the work by Lai et al., a quaternary ammonium chitosan hydrogel containing polydopamine-coated carbon nanotubes and platelet-rich plasma fibers provided bacteriostasis, self-healing, conductivity, and NIR-driven photothermal heating; combined with electrical stimulation, this system significantly enhanced epithelization, angiogenesis, and collagen deposition in complex wounds [106].

Carbon-based nanocomposite hydrogels can thus combine real-time monitoring, antimicrobial action, drug delivery, and self-healing mechanics in a single platform for chronic wounds, burns, and diabetic ulcers [75,76,79,103].

### 4.3. Polymeric Nanomaterials and Nanocomposite Hydrogels

Biopolymer-based nanoparticles and hydrogels (e.g., chitosan, alginate, cellulose, hyaluronic acid, PLGA) mimic the extracellular matrix while offering excellent biocompatibility, biodegradability, and tunable mechanics [94,95,96]. Incorporation of metallic or carbon nanofillers creates nanocomposite hydrogels with:

- Improved mechanical strength, moisture management, and oxygen permeability.
- Enhanced antibacterial, anti-inflammatory, and antioxidant properties.
- Stimuli-responsive and sensing capabilities, such as pH-triggered drug release or built-in optical indicators of infection [75,77,82,94].

In a study by Gamal et al., a bilayer dressing with a 3D-printed alginate/gelatin/PVA hydrogel bottom and a polycaprolactone/cellulose acetate nanofiber top incorporated nanoparticle-based delivery of antioxidant and anti-inflammatory drugs, providing strong antibacterial activity and boosting diabetic wound closure to ~95% at 14 days with improved neovascularization and collagen deposition [107].

In the work of Wu et al., a thermosensitive chitosan–β-glycerophosphate hydrogel loaded with self-assembled nanoparticles (CIZ; chlorogenic acid, indocyanine green, Zn^2+^) offered rapid in situ gelation, combined antioxidative, anti-inflammatory, angiogenic, and photothermal antibacterial effects, and significantly accelerated remodeling of diabetic foot ulcers in vivo [107].

In the study by Yun et al., Thonningianin A nanoparticles were embedded into a thermosensitive chitosan hydrogel, yielding a composite (TA-NPs@Gel) with strong antioxidant and pro-angiogenic activity that markedly improved wound closure in diabetic mice, partly via activation of the HIF-1 signaling pathway [108].

These systems support cell adhesion and angiogenesis and can be engineered for on-demand release of growth factors, antibiotics, or analgesics in response to wound-specific cues [77,82,96,99].

### 4.4. QDs and MXenes

Semiconducting QDs and 2D MXenes are emerging as next-generation transducers. QDs and carbon/graphene QDs offer bright, stable fluorescence for sensitive optical biosensing, enabling multiplexed detection of wound biomarkers and bacteria at low concentrations [75,76,79].

In the work by Shi et al., a bilayer gelatin methacryloyl microneedle patch loaded with polydopamine-coated ceria nanozymes and nitrogen-doped carbon QDs enabled gradient photothermal antibacterial therapy, exudate control, ROS scavenging, and real-time pH monitoring via fluorescence, achieving ~99.9% closure and robust angiogenesis in diabetic wounds [107].

In the study by Wang et al., a lipoic-acid-modified chitosan hydrogel incorporating carbon QDs and ceria–MoS_2_–polydopamine nanoparticles provided reversible, smartphone-readable pH-sensitive fluorescence alongside potent photothermal antibacterial, antioxidant, and anti-inflammatory functions, thereby promoting diabetic wound healing [109].

In the work of Ou et al., a Ti_3_C_2_Tx MXene–silver nanocomposite hydrogel containing paeoniflorin (KC@PF@TA) combined strong photothermal antibacterial properties with controlled bioactive molecule release, yielding significant antibacterial, anti-inflammatory, angiogenic, and tissue-regenerative effects in *S. aureus*–infected diabetic mouse wounds [110].

MXenes (e.g., Ti_3_C_2_Tx) provide high electrical conductivity, hydrophilicity, and photothermal activity, making them attractive for electrochemical sensing, e-skin motion monitoring, and photothermal-assisted antibacterial therapy [77]. MXene-based natural-polymer hydrogels can function as wearable, skin-conformal sensors and, when combined with electrical or photothermal stimulation, have been shown to accelerate wound closure in preclinical models [75,77].

### 4.5. Nano–Bio Interfacial Sensing Mechanisms

A mechanistic understanding of signal generation at the nano–bio interface is essential for evaluating sensing selectivity, sensitivity, and stability. In nanomaterial-enabled wound systems, detection arises from physicochemical interactions that modulate electronic structure, interfacial charge transfer, optical transitions, or dielectric properties. These processes ultimately determine signal-to-noise ratio and long-term robustness in complex wound exudate environments.

#### 4.5.1. Electron-Transfer Kinetics in Electrochemical Sensing

Electrochemical wound sensors operate via heterogeneous electron-transfer reactions governed by Butler–Volmer kinetics and interfacial charge-transfer resistance [111,112]. The electron-transfer rate constant (k^0^) depends on electrode electronic structure, surface area, and adsorption energetics [113,114].

At nanostructured electrodes, increased surface curvature and high density of catalytic sites reduce activation energy barriers, enhancing Faradaic current responses [115].

For carbon nanomaterials, edge-plane defects and oxygen-containing functional groups significantly accelerate electron-transfer kinetics compared to basal planes [116,117]. Similarly, graphene’s conductivity and defect density modulate electrochemical sensitivity [118].

In MXenes such as Ti_3_C_2_Tx, metallic conductivity combined with surface terminations (–OH, –O, –F) influences interfacial charge distribution and redox activity [119,120].

Surface chemistry directly alters charge-transfer resistance and double-layer capacitance, which are critical parameters in impedance-based wound sensing [121,122].

In ROS detection (e.g., H_2_O_2_ oxidation), catalytic nanomaterials lower overpotential and increase current response [123]. While nanomaterials provide superior sensitivity, they often exacerbate biofouling due to their high surface energy and surface area. To mitigate these issues, surfaces are often modified with anti-fouling coatings (e.g., PEG) or designed with porous, anti-fouling architectures [123,124].

#### 4.5.2. Band Structure, Defect States, and Fluorescence Quenching in QDs

In semiconducting QDs and CQDs, sensing behavior is governed by quantum confinement and surface-state-mediated recombination [125,126]. The bandgap energy is size-dependent, while heteroatom doping introduces mid-gap states that modulate electron–hole recombination and fluorescence efficiency [125,126].

ROS detection frequently relies on fluorescence quenching mechanisms described by the Stern–Volmer model [127].

For CQDs, surface defects and oxygen-rich functional groups determine sensitivity to oxidative species [128].

Excessive defect states, however, introduce nonradiative recombination pathways, lowering quantum yield and reducing signal stability [129]. Therefore, surface passivation strategies are critical to maximize signal-to-noise ratio in wound exudate environments [129].

#### 4.5.3. Plasmonic Resonance Shifts in Metallic Nanostructures

Metallic nanoparticles such as Au and Ag support localized surface plasmon resonance (LSPR), where collective oscillations of conduction electrons respond to local dielectric changes. Binding of biomolecules or pH-induced refractive index variation shifts the plasmon resonance peak [130,131].

The magnitude of LSPR shifts depends on nanoparticle geometry, dielectric environment, and surface ligand chemistry [132]. At the wound interface, adsorption of proteins, bacterial toxins, or enzymatic cleavage products modifies the local refractive index, enabling label-free detection [133,134]. However, nonspecific adsorption can introduce background signal shifts, necessitating antifouling surface engineering [135].

#### 4.5.4. Redox Cycling and Nanozyme Activity in ROS-Modulating Systems

Certain nanomaterials (e.g., ceria nanoparticles) exhibit enzyme-mimetic activity through reversible valence changes (Ce^3+^/Ce^4+^ redox cycling) [136,137]. Oxygen vacancy concentration determines catalytic efficiency and ROS scavenging behavior [136,137].

This redox cycling modulates both therapeutic antioxidant activity and electrochemical signal generation [138,139]. However, sustained oxidative stress may alter nanoparticle oxidation state distribution, affecting long-term sensing reliability [138,139]. Understanding defect chemistry and vacancy-mediated charge transport is therefore essential for stable ROS monitoring.

#### 4.5.5. Signal-to-Noise Ratio and Interfacial Stability

Signal reliability in wound sensing depends on minimizing interfacial noise sources such as [140]:

- Biofouling-induced impedance shifts
- Nonradiative fluorescence decay
- Electrode polarization
- Mechanical strain–induced resistance variation

Surface modification strategies such as PEG coatings reduce nonspecific protein adsorption and improve electrochemical stability [135].

## 5. Flexible and Wearable Nanomaterials-Based Nanosensors for Wound Monitoring/Healing

The integration of nanotechnology with wearable and flexible electronics represents a transformative step in wound management. Traditional wound assessment often relies on visual inspection and sporadic biochemical testing, which provide limited real-time information. In contrast, wearable nanosensors offer continuous, non-invasive monitoring of wound physiology, enabling early detection of infection, inflammation, and delayed healing. These systems are particularly valuable for chronic wounds such as diabetic ulcers, venous leg ulcers, and pressure sores, where timely intervention is critical to prevent complications [141,142,143,144,145]. These technologies aim to replace episodic, clinic-based assessment with continuous, data-driven monitoring and tailored intervention at the point of care.

Continuous, on-body monitoring of pH, temperature, moisture, uric acid, oxygen and infection-related metabolites (e.g., trimethylamine) in wound exudate is repeatedly highlighted as a central goal of wearable biosensor research [144,146,147,148]. Reviews emphasize that these markers track inflammation, bacterial burden and tissue regeneration and can guide earlier, more personalized treatment [11,143].

To achieve conformal contact with irregular wound beds and moving skin, most devices rely on flexible, stretchable and/or hydrogel-based platforms, including soft elastomers, conductive hydrogels and textile-integrated sensors [145,149,150,151,152,153]. Such substrates improve mechanical comfort, reduce motion artefacts and allow integration of microfluidics for exudate handling [144,145,146].

### 5.1. Color-Changing/pH-Responsive Systems

pH is among the most widely targeted wound biomarkers: a shift from slightly acidic toward alkaline values often accompanies infection or stalled healing [144,151]. Numerous colorimetric pH-sensing dressings embed pH-sensitive dyes or nanoparticles into hydrogels or fibers; changes can be read by eye or quantified via smartphone image analysis [11,151]. These systems leverage halochromic dyes, fluorescent nanomaterials, or photonic nanostructures, often integrated within hydrogels, electrospun fibers, or textile matrices, to achieve rapid and reversible color responses corresponding to clinically relevant pH ranges.

For example, Banitorfi Hoveizavi et al. fabricated PVA/Gel-A nanofibrous hydrogels doped with bromothymol blue, achieving stable and reversible color transitions between pH 4–10 while minimizing dye leaching through ionic crosslinking [3].

**Figure 5:**
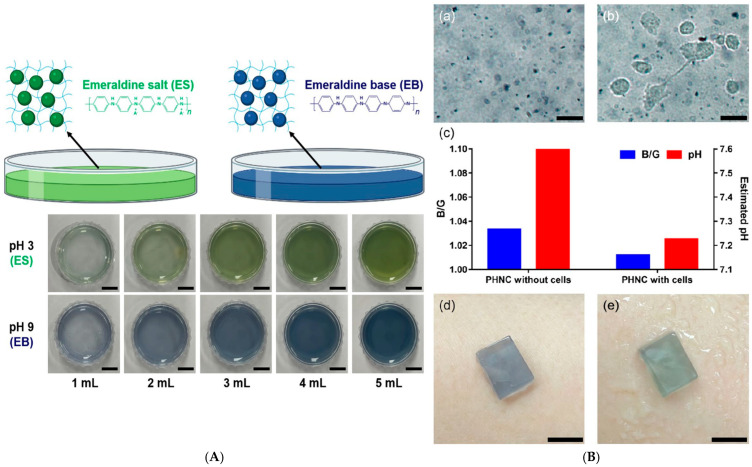
(**A**) Schematic representation of the molecular configuration within PNHCs synthesized at varying pH levels (3 and 9), illustrating the influence of solution pH on their internal structure. Images display PNHC samples prepared with different volumes (1, 2, 3, 4, and 5 mL) under both acidic (pH 3) and alkaline (pH 9) conditions. (**B**) (**a**) PNHC sample without cells and (**b**) PNHC sample after 6 h of cell attachment. (**c**) Blue-to-green (B/G) intensity ratios of PNHCs with and without cells, along with the corresponding pH values estimated using the calibration model shown in (**d**). The B/G ratio was calculated as the blue channel intensity divided by the green channel intensity, with color quantification performed using ImageJ software (Version 1.54p). Photographs of (**d**) PNHC applied to human skin and (**e**) PNHC following exposure to artificial sweat solution. Scale bar = 5 mm [151].

Similarly, Lee et al. developed polyaniline nanoparticle–hydrogel composites (PNHCs) that act as colorimetric pH sensors capable of detecting subtle variations in wound exudate acidity. The developed PNHCs exhibited a highly linear colorimetric response across varying pH levels (Figure 5A), confirming their suitability for precise pH sensing applications. Furthermore, the system successfully detected subtle pH variations induced by metabolites from cancer cells (Figure 5B), demonstrating both high sensitivity and excellent biocompatibility [151].

**Figure 6:**
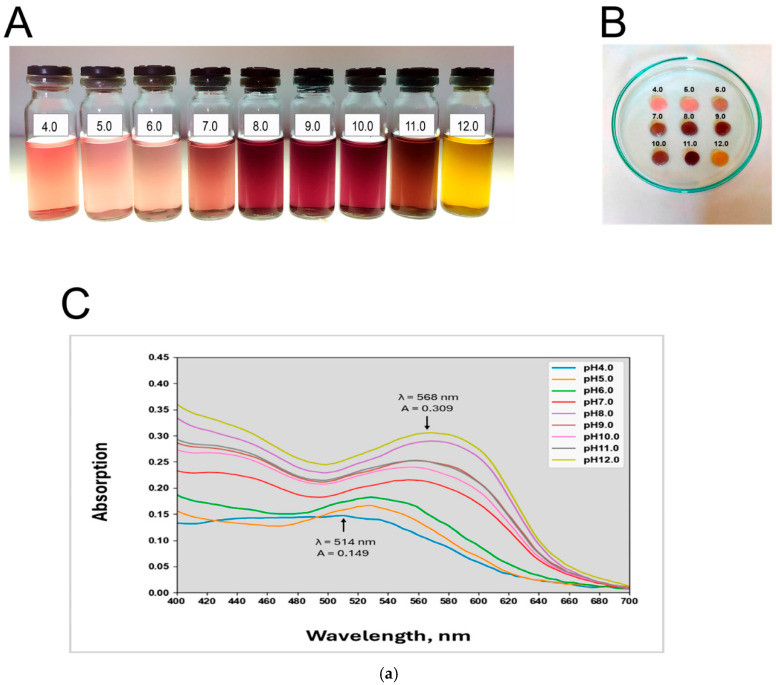

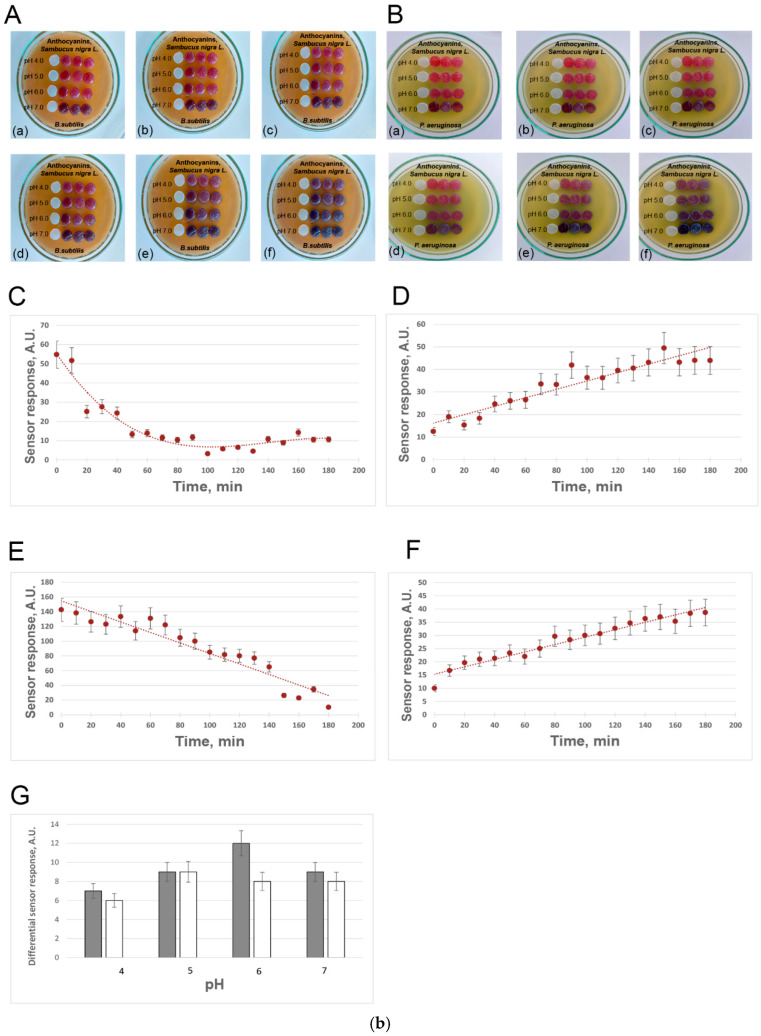
(**a**) (**A**) pH-dependent color variation of elderberry anthocyanin extract (pH 4–12); (**B**) BC hydrogels loaded with anthocyanins across the same pH range; (**C**) corresponding UV–Vis spectra (400–700 nm); (**b**) Smartphone-based colorimetric sensing using anthocyanin-functionalized BC hydrogels. (**A**,**B**) Time-dependent color changes during incubation on *Bacillus subtilis* and *Pseudomonas aeruginosa* lawns and in buffer solutions (0–180 min). (**C**–**F**) Quantified red and blue channel intensity changes over time. (**G**) Differential blue-channel response after 30 min incubation, correlated with immobilization pH and bacterial exposure [152].

An intelligent hydrogel wound dressing based on bacterial cellulose (BC) was developed for monitoring microbial infection through the incorporation of a natural pH-responsive dye, anthocyanins (ANC) extracted from elderberry (*Sambucus nigra* L.). The immobilized anthocyanins enabled sensitive detection of bacterial metabolites, with the highest sensor responses observed at pH 5.0–6.0, which is highly relevant to early-stage wound infection. The limit of detection was determined to be 3.45 A.U., as quantified using a smartphone-based colorimetric analysis application (Figure 6) [152].

**Figure 7:**
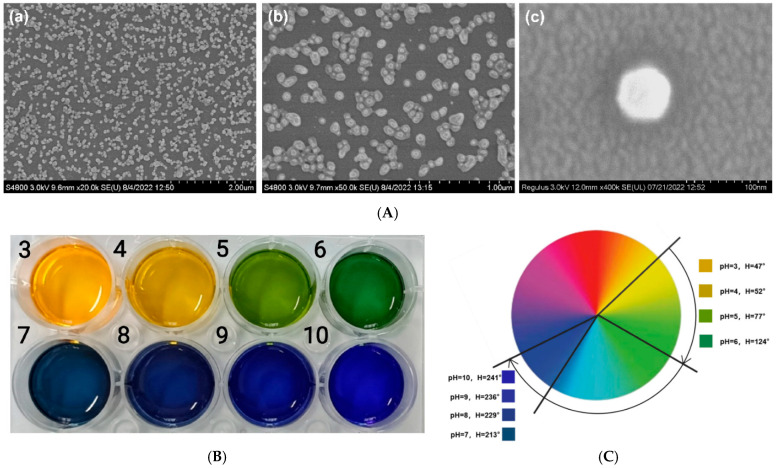
(**A**) SEM images of nanocapsules examined at different configurations: (**a**) 20,000×, (**b**) 50,000×, and (**c**) 400,000×; (**B**) Color of the nanocapsule suspension at pH 3–10 (the numbers represent pH values); (**C**) The H-value range of nanocapsules under different pH values (pH 3–10) [153].

Hou et al. fabricated wide-domain pH color-changing nanocapsules containing thymol blue, bromocresol green, and bromocresol purple, encapsulated in ethyl cellulose and integrated into sodium alginate hydrogel fibers. The system displayed continuous color change from yellow to green to blue (pH 3–10), suitable for wound exudate and sweat monitoring (Figure 7) [153].

In diabetic wound care, Xu et al. developed smart photonic crystal hydrogels (PCHs) capable of visually detecting glucose, pH, and temperature changes simultaneously. The pH response relied on acid-base indicator incorporation, offering real-time monitoring of metabolic and infection-related alterations [154].

Fluorescent and carbon-based nanomaterials offer greater sensitivity and reusability compared to traditional dyes.

For instance, Yang et al. synthesized orange-emissive carbon QDs (O-CDs) that exhibit both colorimetric and fluorescent changes across a wound-relevant pH range (5–9). When immobilized on medical cotton cloth, these O-CDs enabled dual-mode (fluorescence and visible) detection of wound pH without interference from blood or exudate [155].

Kromer et al. reported a fluorescent polymeric nanosensor composed of polystyrene nanoparticles functionalized with fluorescein dye, capable of detecting extracellular pH changes in biofilms via ratiometric fluorescence. Such nanosensors can be adapted for wound biofilm detection [156].

Near-infrared (NIR) fluorescent probes, such as the silica-supported tricarbocyanine nanosensor developed by Terrones et al. extended the functionality for deep-tissue monitoring, minimizing interference from skin scattering [157].

More complex smart composites are now being developed to couple diagnostic sensing with therapeutic action. For instance, He et al. reviewed multifunctional hydrogels responsive to pH, ROS, and glucose, capable of dynamically releasing antimicrobial or angiogenic agents while simultaneously monitoring wound chemistry [158]. Li et al. further showed how integrating wearable hydrogel electronics enables real-time communication between the wound environment and external AI-assisted monitoring systems, creating closed-loop smart bandages [159]. Other examples include hydrogel films doped with dual fluorescent pH dyes for quantitative mapping of wound pH on discarded clinical gauze, pH-responsive fibers and paper bandages for low-cost visual assessment [144,150], and smartphone-readable indicator systems that support remote monitoring and telemedicine workflows [151].

These innovations demonstrate how bioengineered nanomaterials can merge biological specificity with textile flexibility, extending the lifespan and reusability of wound biosensors.

### 5.2. ROS Nanosensing Systems

ROS (including H_2_O_2_, OH, and O_2_^−^) are double-edged mediators in wound repair. At physiological levels, they promote angiogenesis and bacterial defense; however, their overproduction leads to oxidative stress, inflammation, and impaired healing [160]. Therefore, quantitative and responsive monitoring of ROS is critical in advanced wound care systems.

Nanomaterial-based ROS sensors operate through redox-coupled catalytic, photoluminescent, or stimuli-responsive mechanisms. Common approaches include:

- Redox catalysis using peroxidase-like nanomaterials (e.g., CeO_2_, MnO_2_, TiO_2_, ZnO) that react with ROS to produce colorimetric or electrochemical signals [160,161].
- Fluorescence modulation, where oxidative species quench or activate the emission of QDs, CDs, or graphene oxide composites [24,162].
- ROS-triggered degradation or drug release, leveraging polymers or linkers that are selectively cleaved by oxidative stress [160].

#### 5.2.1. ROS-Sensitive Nanomaterial Systems

Metal oxides such as CeO_2_, TiO_2_, and ZnO are known for their intrinsic peroxidase- and catalase-like activities, enabling both ROS generation and scavenging depending on redox conditions [160]. Their dual functionality supports oxidative disinfection and antioxidant balance during different wound-healing stages [160].

For example, CeO_2_ nanoparticles (Ce^3+^/Ce^4+^) can reversibly catalyze H_2_O_2_ decomposition, acting as self-regulating redox nanozymes when embedded in hydrogel wound dressings, allowing real-time oxidative stress control and anti-inflammatory protection [163].

A core–shell CeO_2_@ZIF-8/Au nanozyme experimentally demonstrated peroxidase-like ROS generation for bacterial killing followed by catalase/SOD-like ROS scavenging as the wound microenvironment evolved, leading to reduced inflammation and accelerated wound repair in vivo [164].

A recent advance is the hybrid Ce:Zn “nanozyme” nanoflower (PDA@SNP@Ce:Zn NFs), which integrates photothermal antibacterial activity with nitric oxide (NO) release for synergistic infected-wound healing. This system effectively eradicated MRSA biofilms and accelerated angiogenesis in diabetic rats [165].

Experimental cellular models demonstrated that CeO_2_ nanoparticles upregulate catalase activity and suppress oxidative stress signaling, protecting cells from ROS-induced damage and supporting tissue repair [166].

Similarly, iron oxide nanoparticles (FeO NPs) demonstrate strong peroxidase-like activity for ROS-mediated disinfection and a robust photothermal response under near-infrared (NIR) light. Their synergistic catalytic and thermal activity achieved near-complete bacterial eradication in infected murine wounds, significantly enhancing re-epithelialization [167].

**Figure 8:**
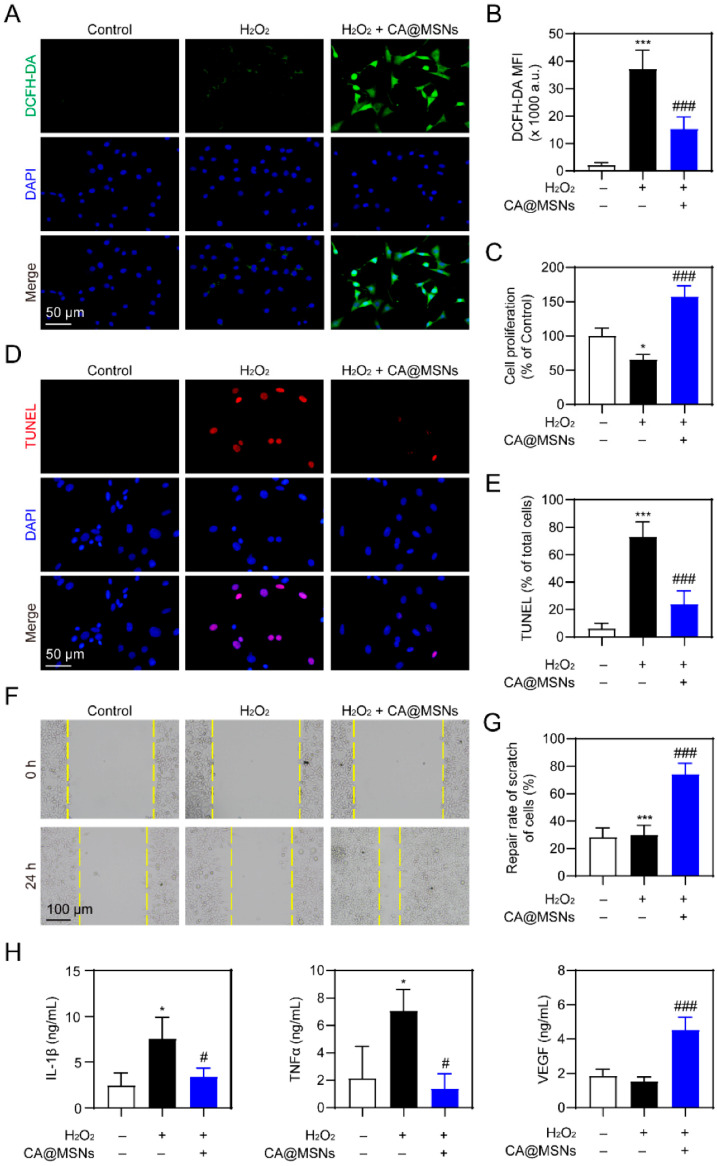
ROS-scavenging and angiogenic activity of CA@MSNs in HUVECs. (**A**) Representative confocal images and (**B**) quantification of ROS levels in HUVECs. (**C**) CCK-8 assay of HUVEC viability with or without CA@MSNs. (**D**) Representative confocal images and (**E**) quantification of TUNEL-positive HUVECs. (**F**,**G**) Cell migration assay (scratch assay) showing the effect of CA@MSNs. (**H**) ELISA quantification of IL-1β, TNF-α, and VEGF levels in HUVEC culture supernatants. * *p* < 0.05, *** *p* < 0.01 vs. Control group; # *p* < 0.05, ### *p* < 0.01 vs. H_2_O_2_ + vehicle group [168].

Yang et al. developed Ce/Ag-doped mesoporous silica nanomaterials (CA@MSNs) that exhibit dual ROS-sensing and ROS-scavenging behavior depending on the wound microenvironment. In inflammatory diabetic wounds, CA@MSNs selectively scavenged excessive ROS, while in infected microenvironments they catalyzed ROS generation to eradicate multidrug-resistant bacteria (Figure 8). This self-adaptive redox behavior functions as a nanozyme-based ROS sensor and regulator, leading to enhanced fibroblast activation, collagen deposition, and accelerated healing in MRSA-infected diabetic mice [168].

Further, MoS_2_-based dual nanozyme hydrogels (MoS_2_@TA/Fe) combine peroxidase and catalase activities to balance oxidative stress and relieve hypoxia, promoting angiogenesis and collagen deposition in infected wounds [169].

Also, Hu et al. reported a Zn-based metal–organic framework (BR@Zn-BTB) nanoparticle system encapsulated within a ROS-scavenging hydrogel. The MOF nanoparticles respond to oxidative stress by releasing Zn^2+^ and berberine, while the surrounding hydrogel neutralizes excessive ROS. The nanocomposite effectively monitors and modulates ROS levels through nanozyme-like activity, leading to reduced inflammation, enhanced collagen deposition, and faster wound closure in diabetic mouse models [170].

ROS-responsive fluorescent and photothermal nanoprobes provide dual functionality: diagnostic readout and therapeutic disinfection.

The MoS_2_–AuAg@PDA composite, integrating both photothermal (PTT) and photodynamic (PDT) functions, generates controlled ROS under NIR irradiation for sterilization while simultaneously sensing oxidative intensity. Its peroxidase-like capacity enables a balanced antibacterial–anti-inflammatory wound microenvironment [5].

Similarly, Mg-doped mesoporous bioactive glass nanoparticles (Mg-MBGs) coated with polydopamine (PDA) and loaded with indocyanine green (MMPI nanocomposite) exhibited low-temperature PTT and ROS-enhanced antibacterial activity, promoting tissue remodeling and macrophage polarization toward pro-healing M2 phenotypes in infected murine wounds [171].

TiCT-Se heterojunction nanosheets (TS) represent another class of ROS-modulating materials. These nanosheets simultaneously exhibit chemical dynamic therapy and PTT. Their catalase- and superoxide dismutase-like activities enable precise ROS regulation, preventing oxidative tissue damage while promoting angiogenesis [172].

Furthermore, Ag-decorated graphene oxide nanoplatforms (Ag/GO-GelMA) have been engineered to enhance photothermal conversion efficiency by over 400%, achieving potent ROS-mediated bacterial killing and downregulation of inflammatory MAPK and PI3K–Akt pathways, thereby accelerating tissue regeneration [173].

#### 5.2.2. ROS-Responsive Drug Delivery Systems

ROS-responsive nanoplatforms are a significant leap forward, and they couple sensing and therapeutic delivery within one device. By leveraging the overproduction of ROS in infected or chronic wounds, these systems selectively trigger the release of antioxidants, anti-inflammatory agents, or growth factors, thereby synchronizing treatment with the wound’s biochemical state [160].

(a) ROS-cleavable polymeric systems

Boronate ester–based polymers and thioketal linkers are widely used in ROS-responsive matrices due to their selective oxidation by H_2_O_2_, which induces cleavage and network degradation. A study by Huang et al. introduced benzene boric acid–grafted polyphosphazene microneedles (PPBA–TP MNs) loaded with green tea polyphenols. Upon exposure to elevated ROS levels in diabetic wounds, these microneedles released antioxidants directly into the tissue, restoring redox balance and accelerating epithelial regeneration [174].

Similarly, Luan et al. developed plant-derived Chinese herbal hydrogel microneedle patches composed of gelatinized starch incorporating aloe vera and berberine. These natural components conferred ROS-scavenging and antibacterial effects, while the hydrogel microneedle matrix provided controlled bioactive release and mechanical integrity, significantly improving infection management and fibroblast proliferation in vivo [175].

In another example, Liang et al. designed a sprayable self-assembled curcumin–metal-polyphenol (TA-CU-CCM) hydrogel network with natural products to reshape the local microenvironment and accelerate the healing process in diabetic wounds. In animal experiments, the sprayable TA-CU-CCM was shown to reshape the wound microenvironment by promoting a shift in macrophages from the pro-inflammatory M1 state to the healing-associated M2 state. This treatment also elevated superoxide dismutase (SOD) activity and increased neovascularization, collectively accelerating wound repair in streptozotocin-induced diabetic mice [176].

(b) Bioactive nanofiber platforms

Electrospun nanofibers serve as ideal carriers for ROS-regulated delivery due to their high porosity, flexibility, and capacity to incorporate redox-sensitive nanocomponents. In this respect, Hajialyani et al. summarized natural product-based nanomedicines (such as curcumin, quercetin, and catechin nanoformulations) which can be integrated in bioactive nanofiber platforms and exert ROS-scavenging, anti-inflammatory, and angiogenic effects through sustained release mechanisms in chronic wound microenvironments [177].

Lv et al. fabricated chitosan–polyvinyl alcohol (PVA) nanofiber mats loaded with the antioxidant ursolic acid, which actively reduced intracellular ROS levels, guided macrophage polarization (M1 → M2), and enhanced angiogenesis in diabetic wounds. Treated animals demonstrated nearly 100% wound closure within 18 days, confirming the therapeutic benefits of redox-regulated nanofibers [178].

In addition, Tiwari and Pathak reviewed multiple local drug delivery strategies for wound repair, emphasizing nanofiber-based and hydrogel composites that combine ROS-sensitive release and antibacterial agents for enhanced skin regeneration [179].

(c) Photodynamic and photothermal nanoplatforms

PDT and PTT nanoplatforms represent a new class of ROS-mediated “on-demand” release systems, where light-induced ROS generation simultaneously disinfects and triggers therapeutic delivery.

Xu et al. developed epigallocatechin gallate (EGCG)-modified black phosphorus QDs (BPQDs) hydrogels for MRSA-infected diabetic wounds. Under near-infrared (NIR) irradiation, the EGCG-BPQDs produced singlet oxygen (^1^O_2_), enhancing bactericidal activity and stimulating angiogenesis. The system achieved 92.4% wound closure within 21 days, outperforming antibiotic-treated controls [180].

Yang et al. synthesized AuAg@PDA–MoS_2_ composites, which leveraged photodynamic and photothermal mechanisms to dynamically manage ROS. In early wound stages, NIR-triggered ROS bursts provided rapid sterilization, while in later phases, MoS_2_’s intrinsic peroxidase-like activity buffered oxidative stress—achieving long-term anti-inflammatory and tissue-repair functions [5].

Miao et al. developed a magnesium-incorporated mesoporous bioactive glass nanoplatform (Mg-MBG–PDA–ICG) exhibiting low-temperature PTT and ROS-enhanced PDT under NIR irradiation. This multifunctional construct eradicated bacterial biofilms, polarized macrophages toward pro-healing M2 states, and upregulated angiogenic markers [171].

Together, these systems exemplify integrated phototherapeutic nanoarchitectures that combine infection control, ROS homeostasis, and regenerative signaling within one modular dressing.

#### 5.2.3. Integrated ROS Sensing and Therapy

The integration of ROS monitoring, modulation, and therapeutic delivery into unified nanoplatforms represents the frontier of smart wound-care technology. These systems exceed passive sensing, evolving into theranostic architectures that actively regulate redox balance, deliver bioactive agents, and promote angiogenesis in situ. The fundamental design principle relies on coupling ROS-responsive nanomaterials (e.g., metal oxides, heterojunctions, or nanozymes) with controlled release and PDT and PTT) component [181,182,183].

Recent reviews consolidate the growing evidence for ROS-integrated nanotherapies. Joorabloo & Liu summarized ROS-scavenging nanozymes and antioxidant nanoplatforms, emphasizing redox homeostasis as the key determinant in regenerative outcomes [184]. Pang et al. outlined intelligent nanomaterial wound dressings integrating ROS detection with phototherapy for infection control and tissue regeneration [181].

A recent advancement was reported by Deng et al., who developed an iron-based catalytic nanozyme (FeNZ) integrated within a metal–organic framework (MOF). The FeNZ system exhibited controllable peroxidase-like activity that could switch between ROS generation and scavenging modes, mimicking a rechargeable battery. This adaptability enabled selective bacterial killing (97.9% for *S. aureus*), followed by suppression of oxidative inflammation during tissue repair, achieving a 95.5% wound-healing rate in infected murine models [185].

Similarly, Cui et al. engineered ROS-modulating TiCT–Se heterojunction nanosheets, which exhibited dual catalase and superoxide dismutase activities. Under NIR light, these nanosheets combined chemical dynamic therapy and PTT, allowing precise ROS modulation, generating radicals for bacterial clearance while scavenging excess oxidative species post-infection. This resulted in significantly reduced inflammation and improved angiogenesis [172].

These dual-function “intelligent nanozymes” illustrate the emerging concept of self-adaptive wound nanoplatforms, capable of autonomous oxidative homeostasis regulation.

A novel generation of redox-regulated nanotherapeutics merges ROS sensing with gene or mRNA delivery.

A ROS-scavenging lipid nanoparticle (TS-LNP)–mRNA formulation was developed to deliver interleukin-4 (IL-4), enabling simultaneous oxidative stress reduction and immunomodulation at the wound site. By eliminating excessive ROS and inducing local IL-4 expression, this platform reprogrammed macrophages from the pro-inflammatory M1 phenotype toward the pro-healing M2 phenotype, thereby enhancing angiogenesis, epidermal regeneration, and overall diabetic wound repair [186].

This gene-augmented nanotherapeutic design exemplifies how ROS-sensitive biomaterials can synchronize immune regulation, oxidative control, and molecular therapy in chronic wound repair.

Integrated gas therapy systems that co-deliver ROS and gasotransmitters (NO, CO) have also shown promise. Padaga et al. introduced a Ce:Zn hybrid metallic nanozyme capable of ROS-regulated nitric oxide release, offering dual antibacterial and pro-angiogenic effects. The nanoflowers eradicated MRSA infections while upregulating VEGF and promoting neovascularization [165].

Similarly, Duan et al. reviewed gas-releasing nanomaterials (e.g., NO, CO, H_2_S donors) as emerging candidates for ROS-regulated wound therapies, emphasizing their spatiotemporal control and antibacterial selectivity [187].

### 5.3. Temperature Nanosensors

Local temperature variation at the wound site is a critical indicator of infection, inflammation, or vascular dysfunction. A localized temperature rise above 38 °C typically signals bacterial colonization or inflammatory response, while abnormally low temperatures can indicate ischemia or poor perfusion. Therefore, real-time thermal sensing provides valuable diagnostic information for early intervention in chronic or infected wounds [86,87].

#### 5.3.1. Thermoresistive Nanosensors

Thermoresistive nanosensors detect temperature variations by translating temperature-induced resistance changes into measurable electrical signals. Their key advantage lies in combining sensitivity, biocompatibility, and mechanical flexibility, allowing real-time thermal mapping of wounds to detect inflammation or infection onset.

MXene (Ti_3_C_2_Tx)-based materials have shown outstanding potential as temperature- and strain-responsive layers in flexible biosensors due to their metallic conductivity, 2D layered structure, and surface hydrophilicity [188].

**Figure 9:**
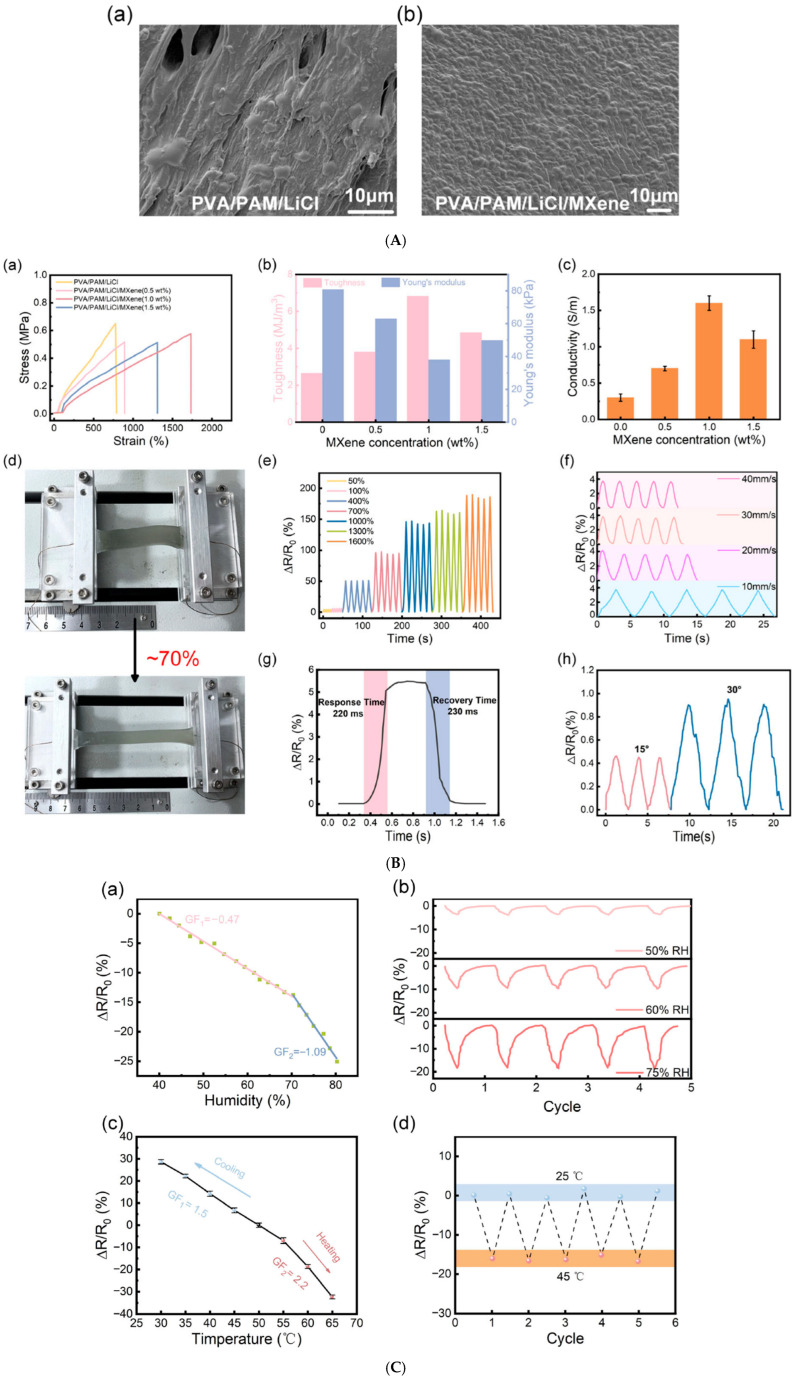
(**A**) SEM images of (**a**) PVA/PAM/LiCl and (**b**) PVA/PAM/LiCl/MXene hydrogels; (**B**) (**a**) Stress–strain curves of PVA/PAM/LiCl/MXene with different MXene concentrations and (**b**) the corresponding toughness and Young’s modulus. (**c**) Effect of MXene concentration on the conductivity of PVA/PAM/LiCl/MXene. (**d**) Physical image of the hydrogel under 70% strain. (**e**) Change curve of ∆R/R_0_ of PVA/PAM/LiCl/MXene hydrogel at different strains. (**f**) Strain curves at different stretching rates under 30% strain. (**g**) Response time and recovery time of the hydrogel under pressure. (**h**) Response at different bending angles. (**C**) (**a**) Humidity sensitivity response curve of the PVA/PAM/LiCl/MXene hydrogel sensor. (**b**) Stability of the hydrogel’s relative resistance change at different humidity levels. (**c**) ∆R/R_0_ curve of hydrogel real-time response to temperature. (**d**) Excellent stability and repeatability after several cycles of hot and cold temperature variations [189].

A study by Li et al. developed a polyvinyl alcohol/polyacrylamide (PVA/PAM)/MXene hydrogel with multifunctional sensing capabilities, including temperature sensitivity (cooling sensitivity = 1.5, heating sensitivity = 2.2) and stretchability up to 1700%. The hydrogel demonstrated fast, reversible thermal response and enabled real-time monitoring of joint movement and local skin temperature; these are key indicators for wound inflammation (Figure 9) [189].

**Figure 10:**
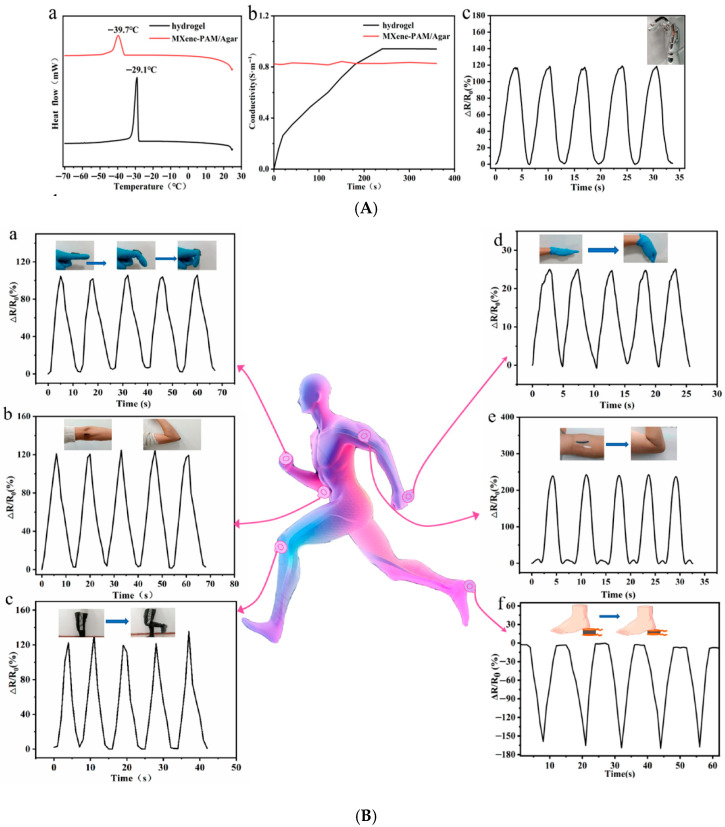
(**A**) Comparison of freezing resistance of the hydrogel and organic hydrogel. (**a**) the DSC curves of hydrogel and MXene-PAM/Agar organic hydrogel showing their crystallization points; (**b**) Conductivity as a function of time after transferring hydrogel and organic hydrogel from a −26 °C refrigerator to room temperature; (**c**) organic hydrogel worked as a wearable strain sensor at −26 °C. (**B**) Real-time monitoring of human motions with finger (**a**), elbow (**b**), knee (**c**), wrist (**d**), and arm muscle movements (**e**). (**f**) The real-time monitoring of foot planter pressure during locomotion [190].

Similarly, Chen et al. designed a dual-network MXene–polyacrylamide/agar (MXene–PAM/Agar) hydrogel that maintained electrical stability and flexibility even at −26 °C, demonstrating adaptability for extreme environments. The hydrogel displayed a wide linear thermosensitive response range and was capable of real-time motion and temperature monitoring (Figure 10), confirming its promise for wearable biomedical devices [190].

**Figure 11:**
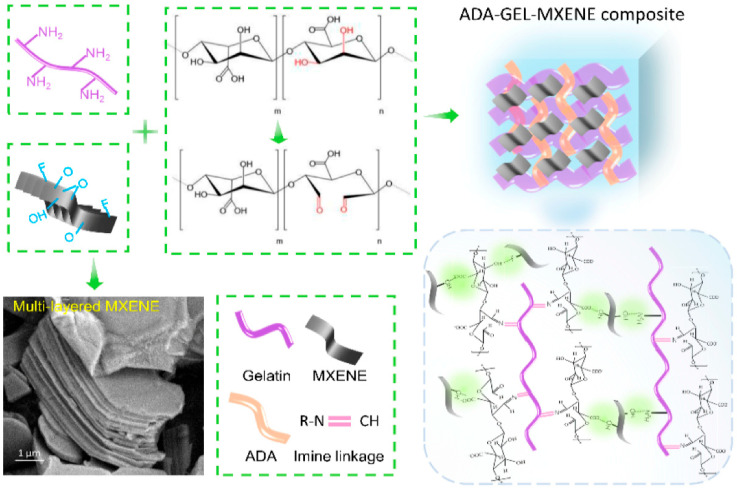
Schematic illustration of the fabrication of composite hydrogel composed of MXene nano-flakes, ADA and gelatin, and the multi-layered structure of MXene nano-flakes [191].

Building on these designs, Zhu et al. synthesized an oxidized alginate/gelatin–MXene (ADA–GEL/MXene) composite hydrogel that combined self-healing, electroactivity, and biocompatibility (Figure 11). The MXene nanosheets enhanced electron transfer and fibroblast attachment, confirming its use for epidermal temperature-sensing and wound-healing applications [191].

Beyond temperature sensing, thermoresistive MXene hydrogels are now multifunctional, integrating thermal feedback, antibacterial activity, and photothermal regulation.

Ou et al. created a Ti_3_C_2_Tx–Ag nanoparticle–Paeoniflorin composite hydrogel (KC@PF@TA) that combined photothermal heating and bioactive molecule release. The hydrogel exhibited real-time temperature monitoring under NIR irradiation and successfully eradicated bacterial infection in diabetic murine wounds while enhancing angiogenesis and fibroblast migration [110].

Additionally, Guo et al. designed a MXene@CeO_2_–gelatin hydrogel with synergistic thermo-photothermal antibacterial and antioxidative functions. The NIR-responsive nanocomposite hydrogel could self-heat for bacterial inactivation and then regulate oxidative stress for wound regeneration, serving as a thermoresponsive theranostic dressing [192].

Graphene remains the most mature nanomaterial for flexible thermoresistive sensing due to its linear resistance–temperature relationship, high carrier mobility, and robust mechanical resilience.

Ding et al. fabricated graphene oxide–coated silk surgical sutures (CA-rGSFS) with a uniform conductive rGO film. These sutures could detect microresistance changes corresponding to temperature or biochemical variations in wound exudates. The system achieved high electrical conductivity (130.3 S/m) and retained performance through 3000 tensile cycles, making it suitable for suture-embedded thermal wound monitoring [193].

In another example, Dou et al. developed a heparin–polydopamine reduced graphene oxide (Hep–PDA–rGO) hydrogel exhibiting 3.63 S/m conductivity, high antibacterial performance, and thermoresistive sensitivity. The hydrogel enabled real-time epidermal temperature mapping while simultaneously reducing inflammation and oxidative stress in chronic diabetic wounds [194].

One of the most advanced developments in thermoresistive biosensing is the bioinspired deformation-insensitive MXene/Clay/PNIPAM (MCP) hydrogel created by Wu et al. By mimicking biological thermoreceptors, the MCP hydrogel maintained stable temperature sensing (~32 °C) regardless of mechanical deformation. This breakthrough enables accurate temperature mapping on dynamic or curved wound surfaces [7].

#### 5.3.2. Thermoelectric and Self-Powered Nanosensors

One of the most promising routes to autonomous wound monitoring lies in thermoelectric nanosensors, which convert local temperature gradients, such as those caused by infection or inflammation, into electrical signals. This allows continuous, battery-free operation and makes these devices particularly suited for long-term, wearable wound applications [195,196].

Yang et al. reported a laser-induced porous graphene foam (LIGF) integrated with thermoelectric components, capable of decoupled strain and temperature detection. The sensor achieved a temperature resolution of 0.5 °C and a Seebeck coefficient of 37 μV/°C, while maintaining 45% stretchability and robustness under deformation. The system successfully enabled self-powered, in situ monitoring of wound-site temperature fluctuations, marking a significant advance toward multimodal wound sensors [195].

Complementarily, Khoso et al. developed reduced graphene oxide (rGO)/PEDOT:PSS nanocomposite-coated textiles that converted low-grade body heat (ΔT = 2.5–16.5 °C) into electrical energy. These wearable thermoelectric generators generated up to 119.5 mV in real human trials, powering small biosensors for health monitoring. The composite maintained conductivity (15 kΩ sheet resistance) even after 20 wash cycles, underscoring its durability for wound-dressing integration [197].

Bismuth chalcogenides, such as Bi_2_Te_3_ and Bi_2_Se_3_, remain the most efficient thermoelectric materials near room temperature. Their integration into hydrogel matrices has enabled self-healing, stretchable, and biocompatible power sources for biomedical use.

Li et al. fabricated a self-healable and stretchable poly(acrylic acid)/xanthan gum/Bi_2_Se_0.3_Te_2.7_ (PAAc/XG/Bi_2_Se_0.3_Te_2.7_) hybrid hydrogel with a Seebeck coefficient of −0.45 mV K^−1^ and an open-circuit voltage of −17.9 mV under a 40 K gradient. Remarkably, after complete mechanical fracture, the hydrogel self-healed within 2 s and retained >99% of its original thermoelectric power output, demonstrating robustness for dynamic wound environments [198].

Hydrogels combined with MXenes or other conductive nanofillers have emerged as soft thermoelectric platforms that maintain electrical responsiveness under deformation.

Zhang et al. reviewed the development of hydrogel-based functional thermoelectric materials, emphasizing MXene–hydrogel composites that leverage high Seebeck coefficients and mechanical softness for biosensing and energy harvesting [199]. Subsequent work by Yu et al. demonstrated a muscle-inspired anisotropic aramid nanofiber aerogel containing MXene and eicosane, achieving Seebeck coefficients up to 46.78 μV K^−1^ and κ = 0.048 W m^−1^ K^−1^. The device could self-monitor and regulate local temperature from 50–400 °C within 1.43 s, showing strong potential for thermal biosensing and smart wound-dressing applications [196].

In a novel biomimetic approach, Geng et al. developed a skin-inspired self-adaptive thermoregulation system based on laser-induced graphene arrays integrated with low-power thermal sensors. The system maintained dynamic temperature control within 35–42 °C, mimicking human thermoreception. Applied to animal wound models, this self-regulating system accelerated healing by ~10% and reduced inflammation by actively maintaining thermal homeostasis [200]. Another work highlights the integration of thermoelectric sensing with closed-loop actuation, bridging passive monitoring and active wound therapy [196].

Zhang et al. recently achieved a record-high room-temperature ZT value of 1.28 in a flexible Ag_2_Se nanowire–graphene oxide–nylon composite film. The system produced an output power density > 9.8 μW cm^−2^ K^−2^ and powered small wearable devices such as thermo-hygrometers and wristwatches. The nylon scaffold provided mechanical flexibility and breathability, underscoring the applicability of Ag_2_Se-based TEGs for bio-integrated wound sensors [201].

#### 5.3.3. Optical and Thermochromic Nanosensors

Optical nanosensors provide non-invasive, real-time visual monitoring of wound status by converting temperature or biochemical fluctuations into colorimetric or fluorescent signals [11,154]. These systems eliminate the need for electronic circuitry and enable rapid, low-cost detection of infection and inflammation, which often manifest as local thermal changes [6]. Optical nanosensors typically rely on thermochromic dyes, fluorescent nanomaterials, or photonic crystal structures, integrated within biocompatible hydrogel matrices that conform to wound surfaces [202,203].

**Figure 12:**
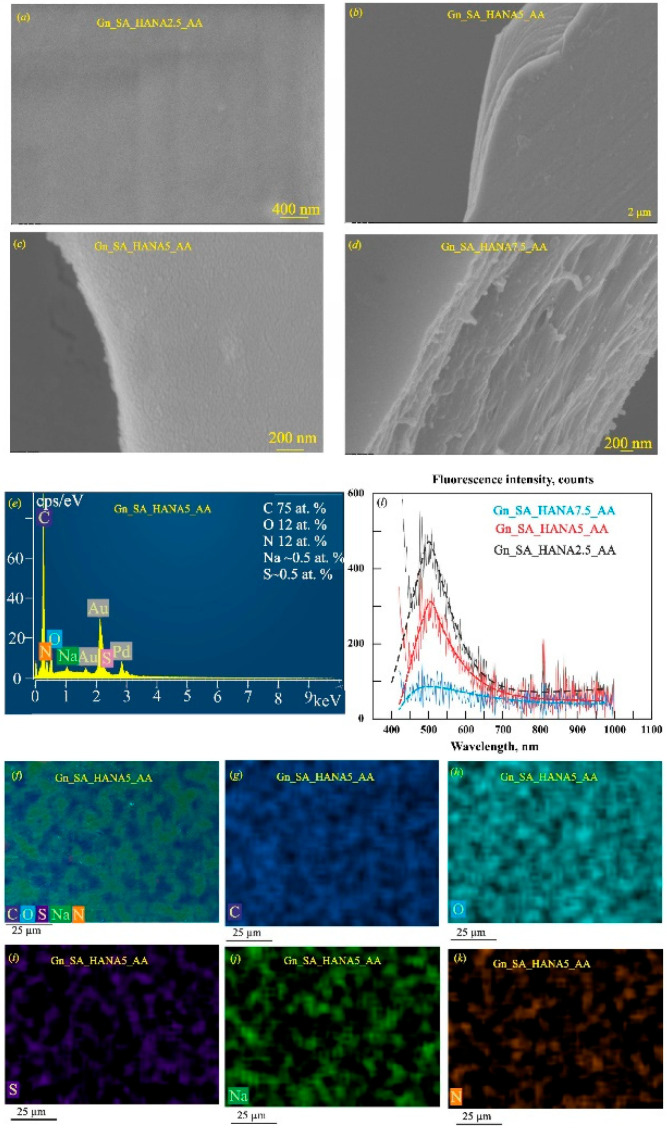
EM micrographs of dried gelatin–alginate hydrogels modified with HANA humic acids and loaded with aminocaproic acid (AA): (**a**) Gn_SA_HANA2.5_AA; (**b**,**c**) Gn_SA_HANA5_AA; (**d**) Gn_SA_HANA7.5_AA. (**e**) EDS spectrum and (**f**) overall EDS elemental map of the dried Gn_SA_HANA5_AA hydrogel. Elemental distribution maps of (**g**) C, (**h**) O, (**i**) S, (**j**) Na, and (**k**) N (all samples coated with a 50 nm Au_80_Pd_20_ conductive layer). (**l**) Fluorescence emission spectra of dried gelatin–alginate hydrogels modified with HANA humic acids and loaded with AA, recorded at an excitation wavelength of 405 nm [204].

Petrushenko et al. introduced a thermochromic, antibacterial, and conductive hydrogel fluorescence from intrinsic alginate and gelatin fluorophores. The hydrogel was modified with calcium and sodium humic acids and was used to monitor the thermal behavior and drug-release capability of these hydrogels. The system enabled controlled delivery of aminocaproic acid through a gel–sol transition near 37 °C, maintaining structural stability and biocompatibility (Figure 12). Optimal formulations containing 2.5–5 wt.% sodium humic acids exhibited a quantum yield of 8–10%, confirming that drug loading preserved the hydrogel’s three-dimensional network and thermosensitivity suitable for physiological conditions [6].

Moreover, Hoveizavi et al. reported colorimetric nanofibrous hydrogels (PVA/Gel-A) loaded with bromothymol blue (BTB), a halochromic dye. The hydrogels achieved visible color shifts (blue–green–yellow) in response to infection-driven pH and temperature changes, with less than 10% dye leaching, highlighting their chemical stability and clinical safety [3].

Fluorescent nanosensors leverage temperature-dependent emission intensity or wavelength shifts for high-sensitivity detection of local thermal fluctuations.

Wang et al. developed fluorescent CDs-functionalized injectable self-healing hydrogels (CD/CxGy). The hydrogels displayed a temperature-dependent fluorescence decrease (~44% after 14 days) as wounds healed, correlating with reduced inflammation and microbial activity. The CD/CxGy platform enabled both visible healing tracking and antibacterial activity, achieving near-complete closure within 14 days in animal models [205].

Furthermore, Ma et al. fabricated Nd–Ca–Si silicate glass/alginate composite hydrogels exhibiting NIR fluorescence intensity linearly correlated with temperature. These materials enabled real-time thermal feedback during photothermal therapy and simultaneously promoted tissue regeneration through bioactive silicate ions [206].

Photonic crystal hydrogels exhibit structural color changes driven by temperature-dependent variations in lattice spacing or refractive index, offering a label-free, quantitative optical approach to wound biomonitoring.

Yang et al. engineered smart photonic crystal hydrogels capable of visual monitoring of glucose, pH, and temperature in diabetic wounds. Temperature sensing was achieved via thermochromic powder integration, providing intuitive color changes that paralleled infection-induced heating. The system enabled simultaneous detection of multiple biomarkers within 15 min [154].

On the fiber-optic front, He et al. created an optical fiber sensor combining poly(allylamine hydrochloride)/SiO_2_ nanoparticles for humidity detection with thermochromic liquid crystal coatings for temperature measurement. The system achieved 3.97 nm/°C sensitivity in the 28–46 °C range, with a 3.1 s response time, supporting its use in minimally invasive, real-time biomedical diagnostics [207].

Beyond passive sensing, new optical materials integrate thermochromic feedback, antibacterial therapy, and fluorescence imaging for comprehensive wound management.

Yang et al. presented a sandwich-structured thermochromic hydrogel patch that exemplifies integrated multimodal wound monitoring and therapy. The hydrogel exhibited temperature-induced color changes, providing visual feedback of wound infection, while the thermochromic response simultaneously triggered controlled photothermal antibacterial activity under external stimulation. In parallel, the patch’s intrinsic electrical conductivity enabled continuous electrical signal monitoring, supporting a closed-loop system that couples real-time sensing with on-demand therapeutic intervention. In in vivo infected chronic wound models, this multifunctional hydrogel demonstrated effective bacterial eradication and accelerated wound healing [6].

Chen et al. advanced this further by engineering dynamic, thermostable peptide-nanoparticle crosslinked supramolecular hydrogels combining thermal responsiveness, anti-swelling stability, and anti-inflammatory wound healing effects [208].

## 6. Multimodal Data Integration and Digital Health Architectures

Importantly, pH, ROS, and temperature biomarkers are physiologically interdependent. Temperature influences enzymatic reaction rates and redox kinetics, thereby modulating ROS production and electrochemical sensing outputs [209]. ROS levels can alter local pH through oxidative reactions and tissue damage, while pH affects redox potentials and nanozyme catalytic activity [210]. During the inflammatory phase, transient ROS bursts and moderate temperature increases may represent normal immune responses, whereas sustained elevations in ROS combined with alkaline pH and hyperthermia are more strongly associated with infection and delayed healing [25,211,212].

Although multimodal sensing of pH, ROS, and temperature improves diagnostic specificity compared to single-parameter monitoring, it introduces substantial technical complexity. Cross-sensitivity between electrochemical, optical, and thermal channels can produce ambiguous outputs if not corrected [25,210,211,212,213,214]. Temperature-dependent Nernstian slope variation affects potentiometric pH sensors, while ROS electrochemical detection is influenced by diffusion and reaction kinetics that vary with temperature [212,213]. Moreover, protein fouling and fluctuating ionic strength in wound exudate can induce baseline drift across multiple sensing modalities [214].

Therefore, the development of reliable multimodal wound monitoring platforms requires advanced signal conditioning, cross-sensitivity correction models, temperature-compensated calibration strategies, energy-efficient hardware design, and robust algorithm validation using clinically representative datasets [214]. Only through integrated systems-level engineering can multimodal pH–ROS–temperature sensing achieves translational reliability in chronic wound management.

### 6.1. Signal Cross-Sensitivity in pH–ROS–Temperature Monitoring

Simultaneous monitoring of pH, ROS, and temperature in wound environments is intrinsically coupled because each parameter influences the electrochemical, optical, or catalytic sensing behavior of the others. These cross-dependencies arise from fundamental thermodynamic, kinetic, and interfacial effects within biofluid-contacting sensors.

#### 6.1.1. Temperature–pH Coupling

Potentiometric pH electrodes follow the Nernst equation, in which the electrode slope (mV per pH unit) is directly proportional to absolute temperature [215,216]. Consequently, infection-induced local hyperthermia can artificially increase or decrease measured pH values if temperature compensation is not applied [215]. In wound environments, where localized temperature elevation may precede visible infection, uncorrected Nernst slope deviations can lead to misinterpretation of alkalinization trends [215].

#### 6.1.2. Temperature–ROS Coupling

Electrochemical ROS detection, particularly amperometric H_2_O_2_ sensing, depends on temperature-sensitive redox kinetics and diffusion coefficients [217,218]. According to Arrhenius-type behavior, increased temperature enhances catalytic turnover and electron-transfer rates, leading to amplified oxidation currents independent of true ROS concentration changes [219,220]. In infected wounds exhibiting hyperthermia, this coupling may falsely suggest elevated oxidative stress unless temperature normalization is implemented [221,222].

#### 6.1.3. pH–ROS Coupling

Redox potentials of many ROS-sensitive nanomaterials, including metal oxides and nanozymes, shift with pH due to proton-coupled electron transfer mechanisms [11,223,224]. Alkaline conditions common in chronic wounds can alter catalytic efficiency, modify ROS scavenging rates, and shift voltammetric peak positions [11,223,224]. Therefore, ROS signal amplitude cannot be interpreted independently of concurrent pH measurement [11,223,224].

#### 6.1.4. Protein Fouling and Exudate Effects

Wound exudate contains proteins, inflammatory mediators, and extracellular matrix fragments that readily adsorb onto sensor surfaces [225,226]. Biofouling alters interfacial impedance, redox peak shapes, optical baselines, and catalytic efficiency, leading to baseline drift and sensitivity degradation [227,228]. Chronic wounds, characterized by high protease activity and oxidative stress, further exacerbate electrode polarization and signal instability [228,229].

Without systematic cross-sensitivity compensation, these interdependent effects may generate false infection alarms or obscure true wound deterioration.

To achieve reliable multimodal interpretation, advanced integration and calibration strategies must be embedded at the hardware and algorithmic levels (Table 3).

### 6.2. Calibration Drift and Long-Term Stability in Wound Environments

Wearable wound biosensors operate in chemically and mechanically aggressive microenvironments characterized by persistent oxidative stress, high protease activity, fluctuating moisture, and repeated mechanical deformation [227,228]. These conditions compromise the long-term stability of pH, ROS, and temperature sensing platforms.

Chronic wounds exhibit sustained oxidative stress and excessive protease activity, which degrade sensor coatings and disrupt electrochemical interfaces [238]. Elevated ROS levels may corrode redox-active electrodes or alter nanozyme catalytic behavior, while protein-rich exudate promotes biofouling that shifts voltammetric peaks, increases interfacial impedance, and reduces sensitivity [227,239]. Similarly, electrochemical ROS sensors experience baseline drift from electrode polarization and temperature-sensitive redox kinetics [227,239].

Mechanical strain further destabilizes sensing systems. Flexible substrates undergo bending and stretching that modify conductive pathways, increase contact resistance, and induce microfractures in nanocomposite films [240,241]. Moisture cycling accelerates hydrogel swelling–deswelling transitions, affecting impedance and thermal readouts [242].

To improve long-term reliability, multimodal systems must incorporate materials-level and algorithm-level stabilization strategies [243]. Integrated thermal channels enable dynamic Nernst correction and normalization of temperature-dependent ROS kinetics. Embedded reference electrodes or stable redox couples provide recalibration baselines to mitigate polarization drift [244,245]. Antifouling coatings such as zwitterionic polymers, PEG, and hydrophilic hydrogels reduce nonspecific protein adsorption and enhance electrochemical stability. Embedded microcontrollers can implement scheduled recalibration cycles and environmental compensation algorithms, while recursive filtering and adaptive threshold models correct gradual signal drift. Machine-learning–assisted approaches, including Bayesian updating and online learning frameworks, show promise for compensating nonlinear degradation patterns during multi-day monitoring [246].

### 6.3. Power Consumption Trade-Offs in Multimodal Wound Patches

Simultaneous monitoring of pH, ROS and temperature significantly increases energy demand compared to single-parameter wound sensors. Multimodal systems require multiple active electrochemical channels, continuous analog-to-digital conversion, embedded signal processing, and wireless data transmission, all of which contribute to elevated power consumption [247,248].

Wearable biosensors operating in remote or outpatient settings must balance sensing fidelity with energy autonomy [247].

Self-powered technologies such as triboelectric nanogenerators (TENGs) and thermoelectric generators (TEGs) have been explored for wearable health monitoring [249,250]. Fluctuating energy harvesting output may introduce noise or signal dropout in electrochemical channels unless buffered through energy storage elements [249,250].

Consequently, purely battery-free architectures remain challenging for continuous multimodal wound monitoring, particularly when high-sensitivity electrochemical detection is required.

### 6.4. AI and ML for Infection Prediction and Clinical Validation

AI–assisted analysis enables advanced pattern recognition across correlated pH–ROS–temperature trajectories, facilitating early detection of wound deterioration and infection before other clinical symptoms appear [251]. Because these biomarkers are physiologically interdependent, machine learning (ML) models can extract nonlinear relationships and temporal patterns that are not evident through single-threshold analysis [252].

Multimodal ML frameworks have demonstrated utility in wearable biosensing and digital health applications, particularly for interpreting complex physiological datasets [253].

The FLEX-AI system by Kalasin et al. used a pH-responsive conductive wound dressing and a deep neural network to classify healing stages and corticosteroid response for chronic skin conditions with ~94.6% accuracy in contactless measurements) [254]. Reviews synthesize multiple examples where wearable sensor streams (pH, temperature, moisture, pressure, biochemical markers) feed AI models that infer healing trajectories and guide individualized care [23,143,147].

The concept of an AI-powered diagnostic patch is now realized in at least two high-profile platforms:

- The PETAL patch is a paper-like, battery-free multiplex sensor with wax-printed microfluidics and five colorimetric sensors (temperature, pH, trimethylamine, uric acid, moisture). Smartphone images are processed by deep learning to classify healing vs. non-healing wounds with up to 97% accuracy in animal models and ex vivo human samples [146].
- The iCares system (Wang et al.) uses a wearable microfluidic device with nanoengineered sensors for ROS, pH and temperature, coupled to machine-learning analysis to classify wound status and predict healing time in patient cohorts [23,149].

Complementary work in surgical-site infections shows that multi-modal sensing of temperature, bioimpedance and oxygen saturation, combined with ensemble machine-learning models, can detect and even predict infection 24–48 h before clinical diagnosis in animal models [255], supporting the idea of early-warning systems rather than purely descriptive monitoring.

Continuous moisture and impedance sensing are used to maintain wounds within an optimal “moisture window”, reducing dressing changes and minimizing maceration or desiccation; impedance-based smart dressings and pressure-ulcer sensors provide quantitative feedback for timely interventions [144].

Finally, several platforms implement highly specific detection of bacterial virulence factors and metabolites, such as pyocyanin from *Pseudomonas aeruginosa*, trimethylamine and uric acid, using electrochemical and colorimetric patches on flexible substrates [144,147]. Reviews present that these metabolite-based sensors can reveal infection hours to days before overt clinical symptoms, positioning them as early-warning components in intelligent wound-care ecosystems [144,148].

These examples substantiate the notion of patch-like “on-skin laboratories” that combine multiplex sensing with AI for decision support.

ML-assisted wound platforms may enable: differentiation between infection and sterile inflammation, early prediction of wound deterioration, identification of abnormal ROS–temperature coupling, modeling of healing phase transitions [256,257].

### 6.5. Closed-Loop Control for pH–ROS–Temperature–Responsive Therapy

True theranostic wound systems aim to integrate multimodal biomarker sensing (pH, ROS, and temperature) with automated therapeutic intervention in a closed-loop framework [258]. Unlike passive smart dressings that merely report wound status, closed-loop platforms dynamically adjust therapy based on real-time biochemical feedback, enabling personalized and responsive wound management [10,259].

**Figure 13:**
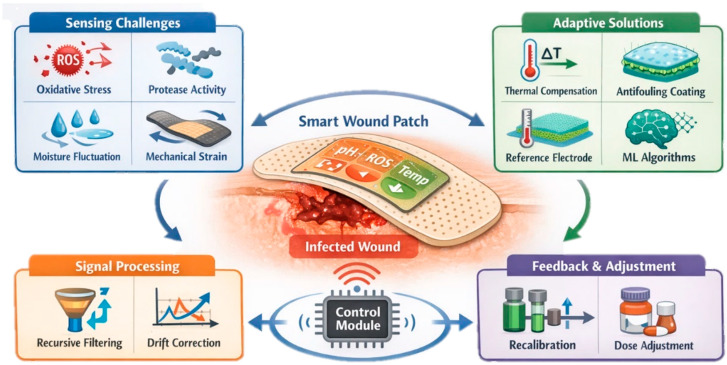
Integrated multimodal closed-loop wound biosensing platform closed-loop system architecture.

The presented schematic illustrates (Figure 13) a next-generation multimodal closed-loop wound monitoring platform designed to simultaneously track pH, ROS, and temperature in a wound environment.

Integrated smart bandages, textiles, and patches now function as wearable on-skin laboratories, coupling multiplex sensing, responsive therapy, and AI-assisted analytics to enable adaptive and personalized wound management. Recent reviews emphasize that theranostic dressings capable of monitoring several biomarkers and executing automated treatment represent a key step toward precision wound care [234].

Mohr et al. developed cellulose-based halochromic textiles functionalized with a 2-hydroxyethylsulfonyl azo dye via vinylsulfonyl conjugation. The system showed fully reversible color transitions (RSD = 0.14%) across pH 5.6–6.9 and was coupled with a miniaturized optoelectronic reflectometer for continuous readout [260].

Mariani et al. introduced a fully textile-based smart bandage with a polymer/iridium oxide pH sensor integrated into an absorbent layer. The device achieved reversible response between pH 6–9 with 59 µA/pH sensitivity and maintained function under fluid flow conditions [261].

Pakolpakçıl et al. developed a halochromic nanofibrous mat by electrospinning alginate and polyvinyl alcohol (PVA) loaded with red cabbage extract, producing rapid color transitions from pink (pH 4–6) to green-blue (pH 7–10), reflecting the acidic-to-alkaline shift during infection [262]. Such natural dye systems exhibit high biocompatibility, rapid response, and eco-friendly fabrication, though their photostability and long-term durability remain limited.

Nanomaterial integration has further advanced this field by enabling enhanced sensitivity, rapid response times, and improved mechanical robustness. Metal oxides such as IrO_2_ and conductive polymers like polyaniline (PANI) have been used as pH sensors within flexible or textile-based substrates. Mariani et al. reported a fully textile bandage with polymer/IrO_2_ pH sensors validated in artificial exudate [150]. These platforms address earlier limitations such as dye leaching and narrow pH ranges [144,151].

Beyond simple colorimetric responses, newer designs integrate fluorescent or genetically engineered biomaterials for more precise, reversible pH sensing.

Saldanha et al. fabricated protein–textile composites embedding pH-responsive fluorescent proteins (pHuji) within amyloid curli fibers, yielding durable, flexible sensors with reversible fluorescence cycles for continuous monitoring [263]. A notable example is the pH-indicating colorimetric hydrogel patch developed by Liu et al. which incorporated phenol red (PR) covalently bonded within an alginate–polyacrylamide (PAAm) hydrogel matrix. The patch changed color from yellow (pH 5–7) to orange/red (pH 7.4–9), matching the clinically meaningful transition between normal and infected wounds [264].

In a study, cotton fabric was cationized using 3-chloro-2-hydroxypropyltrimethylammonium chloride (CHPTAC) and subsequently dyed with curcumin to produce a pH-sensitive textile. The dyed cationized cotton exhibited a roughened surface due to curcumin adsorption and showed distinct, naked-eye-visible color changes with pH, shifting from bright yellow under acidic conditions (pH ≤ 6) to reddish brown in alkaline environments (pH ≥ 8). The curcumin-dyed cationized fabric demonstrated enhanced bactericidal activity compared to untreated and solely cationized cotton, likely due to increased bacterial membrane permeability. No adverse biological responses were observed, and the dyed fabric showed improved anti-adhesion properties, indicating resistance to biofilm formation. The results suggest that curcumin-functionalized cationized cotton is a simple, effective, and biocompatible pH-responsive textile suitable for smart wound dressing application [265].

A dual-sensor hydrogel “GelDerm” was proposed by Mirani et al. Concretely, a hydrogel dressing combines colorimetric pH and glucose sensors with antibiotic and growth factor reservoirs; in murine models, it detects infection, supports diabetic wound management, and improves closure in both infected and non-infected wounds [266]. Pang et al. proposed a UV-triggered hydrogel–electronics hybrid. A bilayer system integrates a temperature sensor and UV-LEDs atop a UV-responsive antibacterial hydrogel; early temperature rise flags infection, and in situ UV irradiation triggers localized antibiotic release, significantly reducing bacterial burden in animal models [148].

Pang et al. combined a flexible temperature sensor and UV micro-LEDs on a soft substrate over a UV-responsive antibiotic hydrogel. Temperature rises indicating infection trigger clinicians to activate UV irradiation, releasing antibiotics on demand and accelerating healing in infected wound models [148].

Xu et al. developed the NFC-powered dressing, which continuously monitors pH, temperature and uric acid and can deliver electricity-controlled antibiotics through a drug-delivery electrode, forming a fully integrated, battery-free closed-loop system [149].

Shirzaei Sani et al. engineered a stretchable, wireless bioelectronic patch that multiplexes electrochemical sensors, antimicrobial release and electrical stimulation for infected chronic wounds, with flexible circuits and on-skin electrodes designed for long-term wear [143].

Ge et al. and related work present wireless smart dressings for exudate management, in which temperature and humidity sensors control local heating to modulate exudate viscosity and promote drug release from hydrogels [23,144].

A range of smart gauze and textile-integrated systems has been proposed. Textile bandages with integrated pH sensors, embroidered electrochemical uric-acid electrodes, and impedance-based pressure and moisture sensors exemplify “gauze that knows when it’s wet/at risk,” supporting early detection of dressing saturation or pressure-ulcer development without removing the cover [144,151].

Curcumin-loaded, pH-responsive bacterial cellulose–poly(acrylic acid) hydrogels modulate release profiles according to wound-like pH, offering controlled antimicrobial therapy fitted to local chemistry [258].

A TPU nanofiber–liquid-metal composite with curcumin-loaded nanoparticles uses NIR-induced photothermal heating for pulsatile drug release, while simultaneously functioning as a strain sensor to monitor wound motion and prevent mechanical re-injury [267].

A microfluidic exudate-managing dressing integrates temperature and humidity sensors with a liquid-metal heater; sensor output governs heating to trigger drug release from a hydrogel, reducing inflammation and enhancing angiogenesis and collagen deposition in infected mouse wounds [268].

Wireless power transfer and energy harvesting are critical for untethered, long-term operation. Jiang et al. developed a wirelessly powered, closed-loop smart bandage with flexible circuits and hydrogel electrodes for impedance and temperature sensing plus programmable electrical stimulation; in mice, this system accelerated healing by ~25% and enhanced dermal remodeling by ~50% compared with controls [269].

Reviews on self-powered body-worn and implantable devices describe energy harvesting from body heat, motion and biofluids and outline how such approaches can underpin future wound sensors and implantable stimulators [270,271,272]. While many bioresorbable, inductively powered devices have been demonstrated primarily for nerve or cardiac interfaces, the same design principles (temporary implants, wireless power, biodegradable substrates) are increasingly discussed for deep wound and tissue-regeneration applications [269,270,271].

Taken together, experimental systems have already achieved fully wireless, closed-loop smart bandages that:

- link via Bluetooth or NFC to smartphones or external readers;
- analyze sensor data in real time; and
- automatically trigger electrical stimulation, heating, or drug release based on pre-set thresholds or algorithmic decisions [146,147,148,149,269].

A demonstration by Xu et al. integrated battery-free, NFC-powered flexible electronics with sensors for pH, temperature and uric acid and an electroactive drug-delivery electrode into a single smart dressing [149]. The near-field communication module harvests power and transmits data to a smartphone, enabling closed-loop infection monitoring and electrically controlled, on-demand antibiotic release in vitro and in vivo [149]. Similarly, SkinAid, developed by Vital et al., demonstrated a textile-based, battery-free smart bandage capable of wirelessly tracking wound pH and uric acid via RF frequency modulation. The system harvested power through a 462 MHz RF signal and retransmitted modulated data up to 2 m, providing continuous and contactless wound tracking [273].

Xu et al. developed a battery-free flexible platform simultaneously measures wound temperature, pH, and uric acid and uses an electronically driven electrode to control antibiotic release, achieving infection suppression and accelerated healing in vivo [274]

Reviews describe a broader ecosystem of battery-free or self-powered systems, including hydrogel-based colorimetric patches (e.g., PETAL) that use energy-efficient imaging plus AI algorithms rather than on-board electronics, and emerging self-powered devices that harvest energy from body heat or biofluids [23,143,147,275]. These strategies reduce device bulk, extend operational lifetime and are especially attractive for home and resource-limited settings [23,147,275]. Additionally, self-powered nanogenerators capable of harvesting biothermal or biofluidic energy are emerging as promising solutions for fully autonomous wound systems [275].

The idea of a fully 3D-printed, battery-free nanomaterial pH sensor aligns with recent printed flexible arrays for pH and bacterial metabolites such as pyocyanin and with NFC-powered smart dressings, even if the exact materials differ. Such platforms demonstrate that fully printed electrodes on flexible substrates can achieve sensitive wound biomarker detection with minimal power budgets [168,169,171].

Beyond pH, flexible organic and inorganic transistor-based sensors and screen-printed electrodes on plastics and textiles have been used to detect uric acid and other analytes in wound fluid, highlighting the uric acid role as a wound-healing and oxidative-stress biomarker) [23,151]. Examples include screen-printed Prussian blue–carbon uric acid sensors integrated into bandages with wireless potentiostats, embroidered gauze sensors, and omniphobic paper-based smart bandages that simultaneously monitor pH and uric acid and report wirelessly [11].

While promising, most current systems remain in preclinical stages. Future development must prioritize scalable design, rigorous validation, and fail-safe engineering to transition from proof-of-concept prototypes to clinically deployable theranostic platforms.

## 7. Challenges and Future Perspectives

### 7.1. Biocompatibility and Long-Term Safety

A primary challenge in translating nanomaterial-based wound biosensors into clinical practice is compliance with standardized biocompatibility frameworks, particularly ISO 10993 testing for medical devices [276,277]. While materials such as graphene, MXenes, metal oxides, and nanozymes exhibit excellent sensing and therapeutic performance, concerns remain regarding ion leaching, nanomaterial accumulation, oxidative cytotoxicity, and inflammatory responses during prolonged exposure [278,279]. For example, although CeO_2_- and MXene-based nanozymes show self-regulating redox activity, their dose-dependent toxicity and degradation behavior in chronic wound environments remain insufficiently understood [164].

Future work must prioritize:

- Full ISO 10993 cytotoxicity, sensitization, irritation, and systemic toxicity panels [276,277]
- Long-term implantation studies
- Biodegradable or bioresorbable nanomaterial designs
- Quantification of degradation products under physiological conditions

### 7.2. Device Classification and Combination Product Regulation

Smart wound dressings integrating sensing, drug delivery, photothermal therapy, and wireless communication may fall under complex regulatory categories. Depending on functionality, these systems may be classified as:

- Class II or Class III medical devices (U.S. Food and Drug Administration—FDA, Silver Spring, MD, USA) [280]
- Active implantable or therapeutic devices (European Medicines Agency—EMA, Amsterdam, The Netherlands) [281]
- Combination products (device + drug + digital health component) [282]

Combination classification significantly increases regulatory burden, requiring [283,284]:

- Preclinical pharmacokinetic and toxicology studies
- Device performance validation
- Human factor and usability testing
- Digital system cybersecurity validation

The integration of ROS-responsive nanozymes or antibiotic release systems further complicates approval pathways, as regulatory authorities may require dual compliance with medical device and pharmaceutical regulations.

Early engagement with regulatory agencies is essential to define approval pathways and reduce translational delays.

### 7.3. Good Manufacturing Practice-Compliant Nanoparticle Manufacturing and Reproducibility

Scalability remains a major barrier towards commercialization [285]. Many nanomaterial-based sensors rely on laboratory-scale synthesis routes with limited batch-to-batch reproducibility. Variability in particle size, surface functionalization, and dispersion stability can significantly affect sensor performance [286].

Translation to good manufacturing practice production requires [287,288]:

- Standardized nanoparticle synthesis protocols
- Tight control of physicochemical parameters
- Cleanroom-compatible fabrication workflows
- In-line quality control for nanocomposite films and hydrogels

MXene synthesis, in particular, involves etching processes that may present reproducibility and contamination challenges at scale. Textile-integrated and screen-printed sensors represent a promising route toward low-cost, disposable smart dressings, but challenges persist in batch uniformity, sterilization compatibility, and regulatory standardization [289].

### 7.4. Sterilization Validation and Device Packaging Constraints

Sterilization compatibility is critical for wound-contacting devices. However, common sterilization methods may compromise nanomaterial performance:

- Gamma irradiation may alter polymer crosslinking density [290].
- Ethylene oxide may affect enzyme activity and surface functionalization [291].
- Autoclaving is often incompatible with hydrogel-based nanocomposites.

Furthermore, packaging must ensure:

- Controlled moisture permeability
- Oxygen exchange
- Electrical insulation
- Mechanical protection without compromising flexibility

Shelf-life validation under accelerated aging conditions is required to ensure stability of sensing elements and therapeutic payloads [292].

Future designs must incorporate sterilization-tolerant materials and validated packaging architectures from early development stages.

### 7.5. Shelf-Life Stability and Environmental Robustness

Wound exudate is chemically complex, containing proteins, enzymes, ions, bacteria, and fluctuating moisture levels [293]. This complexity often leads to cross-sensitivity and signal interference, particularly in electrochemical and optical sensors [294]. For instance, pH and ROS sensors may be affected by ionic strength, protein fouling, or non-specific redox reactions, reducing accuracy over time [87].

Future strategies should focus on anti-fouling coatings, selective surface functionalization, ratiometric sensing, and multimodal signal cross-validation to improve robustness [295,296].

Additionally, devices must demonstrate:

- Storage stability
- Resistance to humidity and temperature fluctuations
- Stable performance after prolonged packaging

### 7.6. Human vs. Rodent Translational Gap

Most reported studies rely on murine diabetic wound models [11]. While valuable for mechanistic insights, these models differ significantly from human chronic wounds skin thickness and structure, immune response complexity, microbiome diversity, healing kinetics, mechanical stress patterns [297].

Large-animal models and randomized human clinical trials remain limited [297]. Without multicenter clinical validation, translation into standard wound care protocols remains uncertain [297].

Future research must prioritize [297]:

- Large-animal validation
- Long-term safety studies
- Standardized outcome metrics
- Head-to-head comparison with conventional dressings

### 7.7. Clinical Trial Design Barriers

Smart wound systems integrating sensing and therapy require complex trial design. Challenges include [228,298,299]:

- Defining clinically meaningful endpoints
- Separating diagnostic from therapeutic benefit
- Controlling for patient variability
- Demonstrating superiority over standard care

Adaptive theranostic platforms must show not only improved healing rates but also cost-effectiveness and workflow compatibility in real-world settings [234].

Closed-loop pH–ROS–temperature wound systems represent a significant step towards precision wound care. However, achieving clinical translation requires [112]:

- Robust cross-sensitivity compensation
- Long-term stability validation
- Energy-efficient actuator integration
- Transparent and explainable decision logic
- Regulatory-compliant safety architectures

### 7.8. Data Privacy, Cybersecurity, and Wireless Compliance (HIPAA/GDPR)

Although self-powered platforms (e.g., thermoelectric and triboelectric systems) represent major advances, stable energy harvesting under low and fluctuating physiological gradients remains challenging. Battery-free devices often produce weak signals that are vulnerable to noise and motion artifacts [195].

Future development should emphasize: (i) hybrid power architectures (thermoelectric + triboelectric + microbattery), (ii) ultra-low-power electronics, and (iii) on-chip signal conditioning and wireless transmission compatible with clinical workflows [300,301].

Most current platforms remain diagnostic or semi-responsive, with limited real-time therapeutic feedback. True closed-loop theranostic wound systems, where sensing directly controls drug release, phototherapy, or electrical stimulation, are still at an early stage. Promising examples include ROS-regulated nanozyme systems and thermochromic–photothermal hydrogels, but these remain largely preclinical [6]. Future platforms must integrate:

- Logic-gated material responses,
- AI-assisted data interpretation, and
- Adaptive therapy algorithms capable of responding dynamically to wound-state transitions [159].

Despite strong theoretical promise, clinical translation of AI-enabled wound monitoring requires rigorous validation frameworks [302]. The FDA has proposed a regulatory framework for AI/ML-based Software as a Medical Device, emphasizing transparency, real-world performance monitoring, and lifecycle oversight [303]. Similarly, evolving European Medical Device Regulation (MDR) standards require clinical evidence and algorithm validation for digital health systems [304].

Wireless-enabled smart bandages introduce digital health considerations. Continuous monitoring systems must ensure [302,303,304]:

- Encrypted data transmission
- Secure authentication protocols
- Compliance with HIPAA (USA) and GDPR (EU)
- Protection against cyber intrusion

### 7.9. Cost–Benefit Considerations and Health System Adoption

For routine clinical adoption, smart wound biosensors must demonstrate:

- Reduced hospitalization time
- Lower infection rates
- Decreased clinician workload
- Clear economic advantage over traditional dressings

High material costs, complex fabrication, and regulatory overhead may limit accessibility. Textile-integrated and printable platforms offer scalable and disposable alternatives, but economic feasibility must be evaluated through health technology assessment frameworks.

### 7.10. Future Perspectives: Toward Intelligent, Closed-Loop Wound Care

The field is moving toward intelligent, patient-specific wound management systems that combine [295,296,305,306]:

- Multimodal sensing (pH, ROS, temperature, oxygen, metabolites),
- ROS- and temperature-responsive nanotherapies,
- Wireless data transmission and AI-assisted decision support.

Emerging directions include gene- and gasotransmitter-integrated nanoplatforms, self-adaptive nanozymes, and digital twin–assisted wound monitoring, which together may enable predictive and personalized therapy [185]

Bridging material innovation with regulatory compliance, scalable manufacturing, clinical validation, and economic viability will determine whether smart wound biosensors transition from promising laboratory concepts to routine clinical tools.

## 8. Conclusions

This review has comprehensively examined recent advances in stimuli-responsive nanomaterial-based biosensor structures for smart wound care, with a particular focus on pH, ROS, and temperature sensing strategies.

A central theme emerging from recent studies is the transition from passive sensing devices to active, theranostic systems. ROS-responsive nanozymes and redox-sensitive polymers not only provide biochemical readouts but also actively regulate oxidative stress and deliver antioxidants or pro-healing agents in response to pathological cues, thereby synchronizing therapy with wound status [164]. Similarly, thermoresistive and thermoelectric nanosensors enable continuous, spatially resolved temperature monitoring, an essential marker of infection, inflammation, and perfusion, while self-powered thermoelectric systems offer a promising route toward battery-free, long-term wound surveillance [188].

Importantly, recent work highlights the value of multimodal and hybrid architectures, in which electrical, optical, and thermal signals are integrated into a single platform. Examples such as thermochromic–conductive hydrogel patches and skin-inspired self-regulating thermal systems illustrate how closed-loop feedback can be achieved, coupling real-time diagnostics with on-demand PTT or pharmacological intervention [6].

Despite these advances, significant challenges remain in terms of long-term biocompatibility, signal selectivity in complex wound environments, scalable manufacturing, and clinical translation. Addressing these limitations will require interdisciplinary collaboration across materials science, bioengineering, electronics, and clinical medicine, as well as early consideration of regulatory and translational pathways.

The intersection of stimuli-responsive nanomaterials, self-powered sensing, wireless communication and intelligent data processing is expected to drive the next generation of wound-care technologies. Such systems have the potential to move beyond reactive treatment toward predictive, personalized, and adaptive wound management. These can ultimately improve the outcomes for patients with chronic, infected, or non-healing wounds. In this context, smart nanomaterial-based biosensors are poised to play a transformative role in the future of precision wound care.

## Figures and Tables

**Table 1:** Key biochemical and physiological markers associated with each stage of wound healing.

Wound Healing Phase	Key Biochemical Markers	Physiological Indicators	Biological Function	References
Hemostasis	Thrombin, Fibrinogen, PDGF, TGF-Î^2^, Calcium ions	Vasoconstriction, platelet activation, pH reduction	Stops bleeding and forms clot scaffold; initiates repair signaling	[16]
Inflammation	Cytokines (IL-1Î^2^, IL-6, TNF-Î±), ROS, pH, chemokines	Localized hyperthermia, erythema, oxygen consumption	Clears debris, fights infection, and activates immune response	[20]
Proliferation	VEGF, FGF, EGF, lactate, glucose	Oxygenation, Temperature stability, Perfusion	Supports angiogenesis, fibroblast proliferation, and collagen synthesis	[20,21]
Remodeling (Maturation)	MMPs, TIMPs, collagen, Uric Acid (UA)	Reduced vascularity, Tissue contraction, pH and oxygen normalization	Remodels ECM, restores tensile strength, and completes tissue maturation	[22,23]

**Table 2:** Wound nanosensors organized by transduction principle.

Transduction Principle	Signal Generation Mechanism	Typical Nanomaterials	Target Parameters in Wounds	Key Advantages	References
Electrochemical	Faradaic current, potential shift, or field-effect modulation upon analyte–electrode interaction	Graphene (FET), CNTs, MXenes, metallic NPs (Au, Ag, ZnO)	pH, glucose, lactate, cytokines (e.g., TNF-α), H_2_O_2_	High sensitivity, rapid response, compatible with flexible electronics	[74,75,76,77,78]
Resistive/impedimetric	Change in resistance or impedance due to adsorption-induced modulation of electron transport pathways	Graphene, CNTs, MXene–hydrogel composites, ZnO, CuO, TiO_2_	Exudate conductivity, bacterial metabolites, hydration level, cell migration	Label-free detection, simple circuitry, strain-tolerant in hybrid composites	[75,76,78,79]
Capacitive/Dielectric	Capacitance variation caused by dielectric constant changes (moisture, ion concentration, pressure)	CNT–PDMS, graphene-coated elastomers, MXene composites	Hydration cycles, exudate accumulation, ion concentration, wound swelling	Low power consumption, easy wireless integration	[80,81]
Optical (fluorescent/plasmonic/colorimetric)	Fluorescence quenching/intensity shift, LSPR wavelength shift, or visible color change due to analyte binding	QDs, carbon dots (CDs), upconversion nanoparticles, Au/Ag NPs, graphene oxide	pH, ROS, MMPs, cytokines, bacterial markers	High sensitivity, visual readout possible, label-free plasmonic detection	[75,78,82]
Piezoelectric/triboelectric	Electric potential generated from mechanical deformation (piezoelectric) or frictional charge transfer (triboelectric)	ZnO nanowires, PVDF, BaTiO_3_, MXene–PDMS composites	Mechanical strain, tissue motion, pressure, deformation	Self-powered operation, suitable for continuous monitoring	[83,84]
Thermal/thermoresistive	Resistance variation or color change induced by temperature fluctuations	Graphene, MXenes, metal nanowires, PNIPAM composites	Local hyperthermia, inflammation, infection monitoring	Rapid thermal response, can trigger temperature-responsive drug release	[85,86]
Magnetic/acoustic	Magnetic field fluctuation (magnetoelastic/magnetoresistive) or ultrasound wave reflection changes	Fe_3_O_4_, CoFe_2_O_4_ nanoparticles, piezoelectric nanocrystals	Mechanical stress, swelling, tissue density, vascularization	Wireless readout possible, non-contact monitoring	[87,88]

**Table 3:** Data fusion and calibration strategies for multimodal pH–ROS–temperature wound monitoring systems.

Strategy	Principle	Relevance to pH–ROS–Temperature Wound Monitoring	References
Ratiometric correction	Dual-channel electrochemical or optical systems normalize analyte signal against a reference channel to reduce environmental bias.	Differentiates true ROS elevation from temperature-induced signal amplification; minimizes photobleaching and intensity fluctuation in optical wound sensors.	[156,230,231]
Temperature-compensated pH calibration	Real-time correction of potentiometric slope using integrated temperature measurements based on Nernst equation adjustments.	Prevents misinterpretation of pH shifts during infection-induced hyperthermia; ensures stable Nernstian response in fluctuating wound environments.	[10,232,233]
Multivariate regression and cross-sensitivity matrices	Mathematical models quantify interdependence between temperature, pH, and ROS signals to compensate for environmental interference.	Enables correction of temperature-amplified ROS currents and pH-dependent redox shifts; particularly relevant in protein-rich, ionically variable wound exudate.	[10,226,234,235]
Principal Component Analysis (PCA)	Dimensionality reduction technique identifying correlated biomarker patterns within high-dimensional sensor datasets.	Distinguishes infection-driven sustained ROS + hyperthermia from transient inflammatory responses; separates multimodal biomarker signatures across healing phases.	[236,237]

## Data Availability

The original contributions presented in this study are included in the article. Further inquiries can be directed to the corresponding author.
